# Embedded Hybrid‐Dimensional Heterointerface for Filament Modulation in 2D Material‐Based Artificial Nociceptor

**DOI:** 10.1002/advs.202401946

**Published:** 2024-08-05

**Authors:** Chang‐Hsun Huang, Te‐Yu Cheng, Chia‐Yi Wu, Kuan‐Hung Chen, Tian‐Li Wu, Yi‐Chia Chou

**Affiliations:** ^1^ Department of Materials Science and Engineering National Taiwan University Taipei 10617 Taiwan; ^2^ Institute of Physics National Yang Ming Chiao Tung University Hsinchu 30010 Taiwan; ^3^ International College of Semiconductor Technology National Yang Ming Chiao Tung University Hsinchu 30010 Taiwan

**Keywords:** 2D materials, artificial nociceptors, gallium oxide, heterointerface engineering, liquid metal printing

## Abstract

Nociceptors are key sensory receptors that transmit warning signals to the central nervous system in response to painful stimuli. This fundamental process is emulated in an electronic device by developing a novel artificial nociceptor with an ultrathin, nonstoichiometric gallium oxide (GaO_x_)‐silicon oxide heterostructure. A large‐area 2D‐GaO_x_ film is printed on a substrate through liquid metal printing to facilitate the production of conductive filaments. This nociceptive structure exhibits a unique short‐term temporal response following stimulation, enabling a facile demonstration of threshold‐switching physics. The developed heterointerface 2D‐GaO_x_ film enables the fabrication of fast‐switching, low‐energy, and compliance‐free 2D‐GaO_x_ nociceptors, as confirmed through experiments. The accumulation and extrusion of Ag in the oxide matrix are significant for inducing plastic changes in artificial biological sensors. High‐resolution transmission electron microscopy and electron energy loss spectroscopy demonstrate that Ag clusters in the material dispersed under electrical bias and regrouped spontaneously when the bias is removed owing to interfacial energy minimization. Moreover, 2D nociceptors are stable; thus, heterointerface engineering can enable effective control of charge transfer in 2D heterostructural devices. Furthermore, the diffusive 2D‐GaO_x_ device and its Ag dynamics enable the direct emulation of biological nociceptors, marking an advancement in the hardware implementation of artificial human sensory systems.

## Graphical abstract

An artificial nociceptor is developed utilizing ultrathin 2D‐GaO_x_, featuring low power consumption and quick switching. Electron energy loss spectroscopy reveals that, when a threshold bias is applied, Ag atoms disperse in the active layer, and then reassemble after bias removal, in order to reduce interfacial energy. The heterointerfaced 2D‐GaO_x_ nociceptor ensures stable, precise charge transfer control, enabling long‐term atmospheric use.

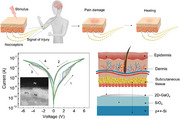

## 1 Introduction

Extensive research has been conducted on 2D materials. Some of these materials have demonstrated high thermal conductivity,^[^ ^1^, ^2^ ^]^ unique electrical–optical properties,^[^ ^3^, ^4^, ^5^ ^]^ strong physical coupling,^[^ ^6^, ^7^ ^]^ and high elastic moduli.^[^ ^8^, ^9^ ^]^ These properties are vital for creating flexible and thermoelectric devices, particularly those that may be used in harsh environments.^[^ ^10^, ^11^ ^]^ However, the mass production of low‐cost, flexible electronic products necessitates a straightforward and efficient approach for the deposition and patterning of these materials. Various techniques have been developed for isolating and fabricating 2D materials.^[^ ^12^, ^13^ ^]^ The most straightforward method is using tape to mechanically extract atomic sheets from a lamellar crystal.^[^ ^14^, ^15^ ^]^ This produces flakes with high crystallinity but a poor yield. Furthermore, the sheets have irregular shapes and lengths in the order of tens of microns, rendering them unsuitable for large‐scale practical applications.^[^ ^16^ ^]^ Chemical vapor deposition (CVD) has significantly progressed as a technique for growing 2D materials.^[^ ^17^, ^18^ ^]^ This technique enables the deposition of materials onto a stiff substrate, such as sapphire or silica, over an area of tens of square centimeters.^[^ ^19^, ^20^ ^]^ However, it typically requires high temperatures and has slow growth rates, making large‐scale manufacturing challenging.^[^ ^21^ ^]^ Liquid metals have many interesting surface and bulk characteristics,^[^ ^22^, ^23^ ^]^ and their use in microfluidic components,^[^ ^24^, ^25^ ^]^ sensors,^[^ ^26^, ^27^ ^]^ soft electronics,^[^ ^28^, ^29^ ^]^ and biomedical disease therapies^[^ ^30^, ^31^ ^]^ has gained considerable interest. A 2D atomic‐scale thin oxide layer spontaneously develops on the surface of liquid metals under atmospheric conditions.^[^ ^32^ ^]^ Liquid metals have a broad range of melting points. They can be used to create diverse alloys with surface properties that can be further modified to fabricate various 2D‐oxide‐based films.^[^ ^33^ ^]^ However, these 2D‐oxide films have not yet been used to produce functional bio‐inspired devices, necessitating more comprehensive research on their electrical properties.

In the human body, nociceptors are vital sensory receptors that recognize external harm and transmit pain signals to the central nervous system.^[^ ^34^ ^]^ They detect noxious (potentially harmful) stimuli, such as excessive heat, stress, and chemical concentrations, and produce pain signals to protect the body.^[^ ^35^ ^]^ Nociceptors typically exhibit threshold and relaxation behaviors.^[^ ^36^ ^]^ “Threshold” behavior indicates reacting to stimuli exceeding a threshold value. By contrast, “relaxation” behavior involves responding to stimuli below this threshold. Moreover, to protect a wounded region, nociceptors may be sensitized after receiving intense noxious stimuli, resulting in the augmentation of their receptivity by reducing their thresholds (allodynia) and amplifying their signals (hyperalgesia).^[^ ^37^ ^]^ This increased responsiveness to external stimuli, while being sensitized effectively, signals injury to the body, prompting a response that prevents further exposure to potentially harmful stimuli.

The development of artificial nociceptors is closely related to advancements in electronic skins. Synthetic nociceptors can be integrated into bionic limbs, increasing their resemblance to natural anatomical structures.^[^ ^38^, ^39^ ^]^ However, advanced materials and electronic devices are necessary to replicate nociceptor functions. Recently, artificial nociceptors based on titanium dioxide,^[^ ^40^ ^]^ Ag‐doped silicon oxide (SiO_x_),^[^ ^41^ ^]^ and zinc oxide^[^ ^42^ ^]^ have been developed. Replicating the intricate nociceptor system with conventional metal–oxide semiconductors necessitates complicated circuitry and high power; thus, this approach is unsuitable for applications requiring energy efficiency.^[^ ^43^ ^]^ The electronic properties of the functional matrix and interfaces of a nociceptor primarily influence its transport characteristics.^[^ ^44^ ^]^ Thus, systematic investigations are necessary to realize nociceptive devices with tunable diffusive dynamics. The qualitative synaptic functionality of some memristors, achieved through thermal dissipation or mobility decay, has been demonstrated and observed to approximate the Ca^2+^ dynamics of chemical synapses.^[^ ^45^, ^46^ ^]^ However, diffusive nociceptors optimized for sensor applications exhibit switching and volatility behaviors that do not precisely replicate the plasticity of natural nociceptors, and the threshold‐switching mechanism of them are not clearly elucidated and observed yet. Accordingly, the development of an artificial nociceptor using 2D‐based ultrathin devices provides insights into investigating the conduction mechanism and advancing 2D bioinspired devices. This innovation enables the creation of a scalable, ultralight, and energy‐efficient nociceptive system suitable for various applications in humanoid robotics. Although artificial nociceptors have advanced significantly in recent years, the specific effects of interfacial active layers with low‐dimensional materials have not been comprehensively studied. Notably, the use of 2D‐gallium oxide (GaO_x_) in bioinspired applications has not yet been explored.

In this study, we employed a liquid metal printing technique to manufacture nociceptive 2D‐GaO_x_ and experimentally investigated a vertical 2D‐GaO_x_ nociceptor device. Experiments were performed to evaluate the properties and behaviors of artificial nociceptors produced using heterointerface engineering. Moreover, to understand the threshold‐switching mechanism of the device, the structural properties and electrical configurations of the 2D‐GaO_x_ were comprehensively analyzed through high‐resolution transmission electron microscopy (HRTEM) and electron energy loss spectroscopy (EELS). The results of this study provide insights into the applicability of 2D‐GaO_x_ in artificial human sensory systems.

## 2 Results and Discussion

### 2.1 Morphology and Characterization of 2D‐GaO_x_

**Figure 1:**
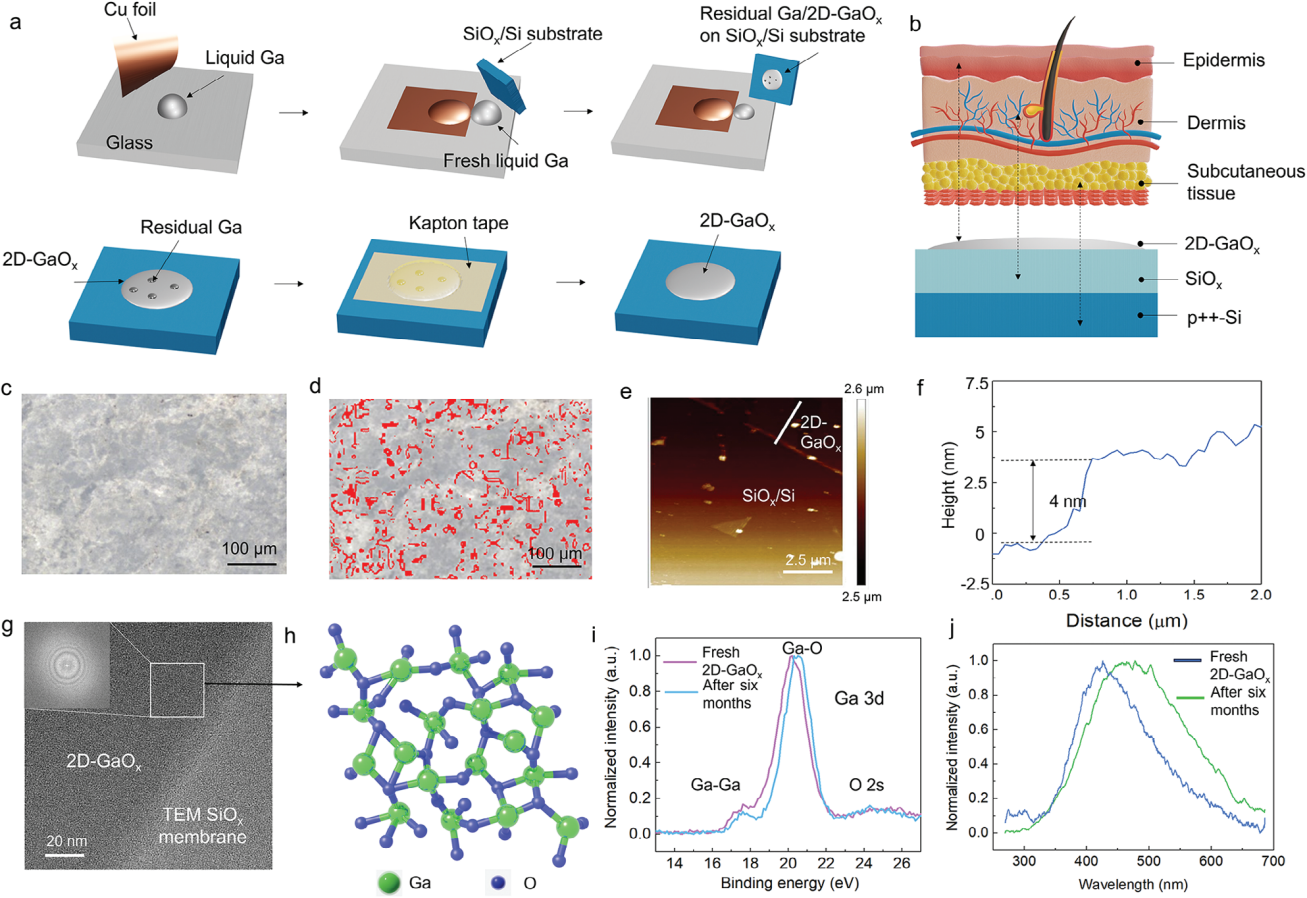
Production and characterization of a large‐area 2D‐GaO_x_ film. a) Liquid metal printing of 2D‐GaO_x_ and efficient removal of residual Ga. b) Correspondence between the structures of human skin and the developed 2D‐GaO_x_/SiO_x_/Si structure. c) Optical image of the surface and d) and an image converted using the Image J software revealing residual Ga (red dots). e) AFM image of 2D‐GaO_x_ on the SiO_x_/Si substrate after removing residual Ga. f) Height profile from the AFM image in (e). g) High‐magnification TEM images of 2D‐GaO_x_ printed on the TEM SiO_x_ membrane. h) Crystal structure of liquid metal‐printed amorphous 2D‐GaO_x_. i,j) XPS Ga 3d and photoluminescence spectra of a 2D‐GaO_x_ film printed on SiO_x_/Si substrate, respectively.

**Figure** **1**a illustrates the process of printing 2D‐GaO_x_ on a SiO_x_/Si substrate. The resulting 2D‐GaO_x_/SiO_x_/Si structure corresponds to the epidermis/dermis/subcutaneous tissue structure of human skin, as shown in Figure 1b. The printed 2D‐GaO_x_ weakly adhered to the Si substrate (Figure S1, Supporting Information), hindering the formation of large‐area 2D‐GaO_x_. The inclusion of a SiO_x_ layer was critical for improving the adhesion of 2D‐GaO_x_ on the Si substrate. During the printing of a 2D‐GaO_x_ film onto a SiO_x_/Si substrate, residual Ga may exist owing to the persistent adsorption force between the liquid Ga and GaO_x_ layer. To ensure that only the 2D‐GaO_x_ film remained on the SiO_x_/Si substrate, heat‐resistant tape was used to repeatedly lift off the residual black Ga (Figure 1c). The residual Ga was efficiently removed, as evidenced by the minimal residue indicated by the red dots in Figure 1d. Figure 1e shows the surface morphology of the 2D‐GaO_x_ obtained through atomic force microscopy (AFM) after eliminating residual Ga from its surface. The height profile of the AFM image (Figure 1f) confirmed the successful production of a large‐area 2D‐GaO_x_ film, with a thickness of ≈4 nm and a root‐mean‐square roughness of 0.582 nm. The fabrication process allows for the printing of large‐area 2D‐GaOx, achieving dimensions up to 0.5 x 0.5 cm on a SiO_x_/Si substrate (Figure S2, Supporting Information). The TEM images indicated that the printed 2D‐GaO_x_ had a continuous film structure (Figure 1g) and an amorphous crystal structure (Figure 1h). A lower‐magnification TEM image confirmed that the printed 2D‐GaO_x_ exhibited a homogenous continuous film without residual Ga (Figure S3, Supporting Information).

X‐ray photoelectron spectrometry (XPS) spectra for Ga 3*d* and O 1*s* were measured to analyze the material characteristics and stability of 2D‐GaO_x_; Figures 1i and S4 show the results, respectively. These figures show slightly left‐shifted binding energy after long‐term storage of the film in the air. The deconvoluted XPS spectrum of Ga 3*d* (Figure S5, Supporting Information) reveals major peaks at 18, 20, 21, and 25 eV, corresponding to Ga–Ga, Ga–O, Ga_2_–O, and O 2*s*, respectively. The O 1*s* XPS spectra were convoluted, revealing three peaks at binding energies of 531.5, 533.1, and 534.9 eV, corresponding to Ga–O, Si–O, and oxygen vacancies (O_vac_), respectively. The ratio of Ga_2_–O to O_vac_ in the 2D‐GaO_x_ layer increased after a 6‐month storage period in the air; thus, the change in the number of O_vac_ in the printed 2D‐GaO_x_ was attributable to phase transitions and deformation during atmospheric storage.

Figure 1j shows the photoluminescence spectra of the samples. Only one broad emission band was detected, with a smaller UV luminescence band at 350–400 nm, broad blue luminescence (BL) band at 400–480 nm, and green luminescence (GL) band at 500–570 nm. The BL band was attributed to the recombination of free electrons with self‐trapped holes and multilevel donor–acceptor pair transitions.^[^ ^47^ ^]^ The GL band was ascribed to the V_Ga_ involved in the 2D‐GaO_x_.^[^ ^48^ ^]^ The BL band broadened after long‐term storage in air, presumably owing to a higher O_vac_ concentration and Ga^3+^ capturing electrons from air. Moreover, the redshift of the luminescence center indicated a shallow trapped electron energy level,^[^ ^49^ ^]^ consistent with the XPS results. These results confirmed the production of a large, homogeneous 2D‐GaO_x_ through liquid metal printing technology, with minor degradation during storage in the air.

### 2.2 Switching Characteristics of 2D‐GaOx Devices

**Figure 2:**
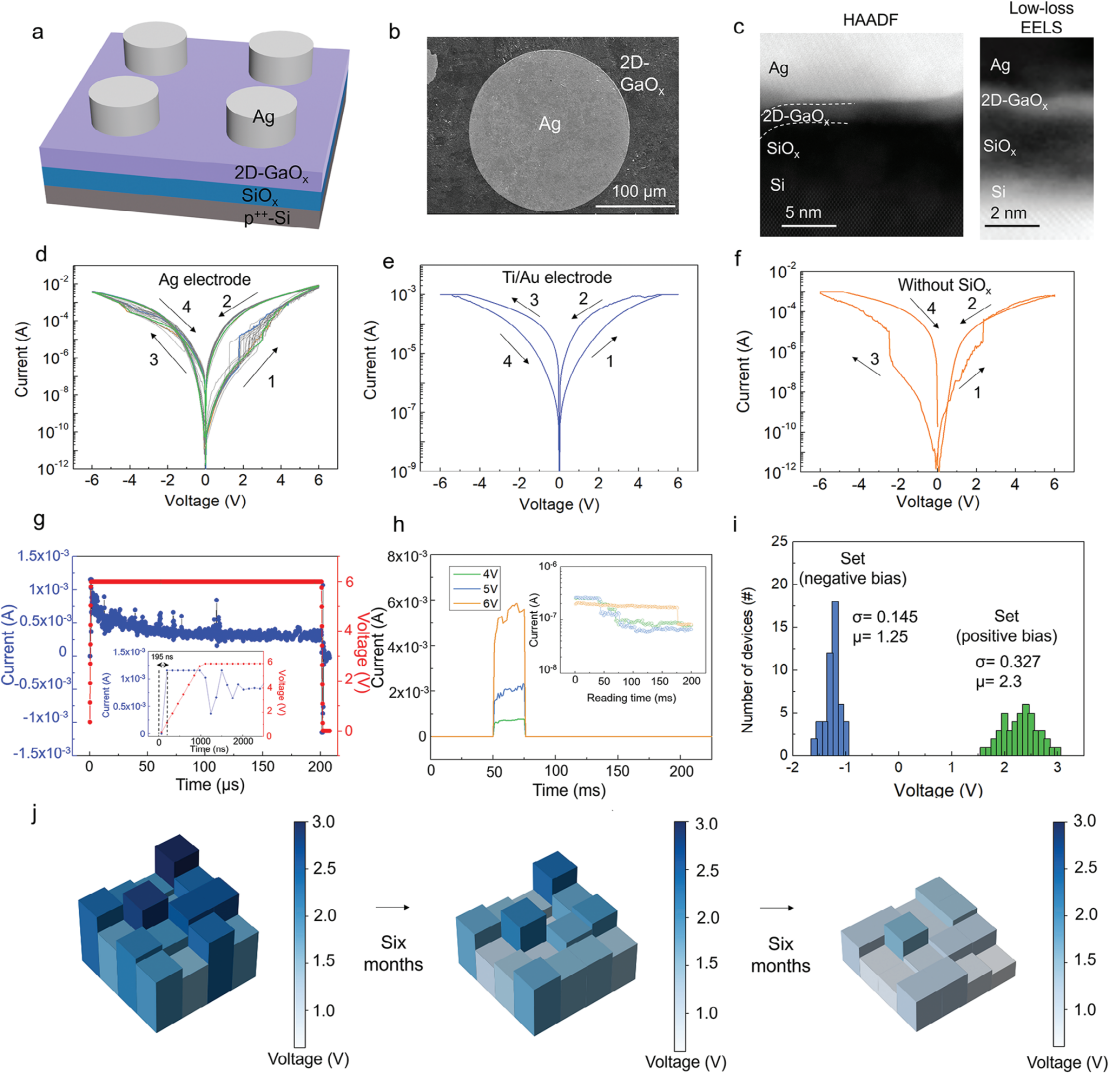
Structure and switching characteristics of the 2D‐GaO_x_ nociceptor. a) 2D‐GaO_x_ nociceptor structure. b) Top‐view SEM image of the Ag electrode on the 2D‐GaO_x_ nociceptor. c) STEM‐HAADF and low‐loss EELS images of the Ag/2D‐GaO_x_/SiO_x_/Si nociceptor. d) *I–V* curves revealing threshold switching under 25 DC voltage sweep cycles. e,f) *I–V* curves of DC voltage sweeps in the device with Ti/Au electrodes and without the SiO_x_ layer, respectively. g) Switching time of the 2D‐GaO_x_ device at the threshold voltage. The red and blue curves denote the input pulse and output current, respectively. The inset shows the enlarged chart for 0–2500 ns switching time. h) Latency characteristics of the device. Inset: Relaxation behavior for 4–6 V input voltages. i) Threshold voltage distributions of 40 2D‐GaO_x_ devices with positive and negative biasing. j) Statistical diagrams of the changes in the positive threshold voltage of 16 2D‐GaO_x_ devices after storage at room temperature for 12 months.

**Figure** **2**a shows a diagram of a vertical two‐terminal artificial nociceptor based on 2D‐GaO_x_. Figure 2b,c shows the top‐view scanning electron microscopy (SEM) images and cross‐sectional HRTEM images of the fabricated 2D‐GaO_x_ device, respectively. The low‐loss EELS image in Figure 2c confirmed the favorable insertion of 2D‐GaO_x_ between the Ag electrode and SiO_x_ layer.

The Ag/2D‐GaO_x_/SiO_x_/Si device was connected to an electrical pulse source for conversion to an artificial nociceptor. Twenty‐five current–voltage (*I–V*) cycles were evaluated to determine the threshold‐switching characteristics of the 2D‐GaO_x_ device (Figure 2d). Initially, the device had a high resistance; however, the current increased when the threshold voltage was exceeded, indicating that the device had transitioned to a low‐resistance state owing to the creation of a conduction path through 2D‐GaO_x_ and SiO_x_. Reducing the voltage caused the device to return to a high‐resistance state, resulting in a current loop. The loop directions were similar for the negative and positive voltage biases. Moreover, the retention test revealed a small disturbance window between the low‐ and high‐resistance states (Figure S6, Supporting Information). These results demonstrated the high volatility and consistency of the Ag‐electrode 2D‐GaO_x_ device, making it suitable for artificial nociceptor applications.

Various *I–V* tests were performed to investigate the effects of the different structural elements on the threshold‐switching characteristics of the device. First, a 2D‐GaO_x_ device with Ti/Au rather than Ag electrodes (Figure 2e) was investigated. Rather than exhibiting threshold nociceptor switching behavior, this device exhibited bipolar resistive switching behavior when the voltage was over 6 V. Similarly, an Ag/2D‐GaO_x_/Si structure (i.e., no SiO_x_ layer) exhibited unstable threshold switching (Figure 2f). This was attributable to the poor adhesion between the 2D‐GaO_x_ and Si, resulting in current leakage after biasing. This indicates that the SiO_x_ layer effectively enhanced the adhesion of 2D‐GaO_x_ to the substrate and its threshold‐switching stability. An Ag/SiO_x_/Si device (i.e., no 2D‐GaO_x_) was also fabricated; its switching behavior included a memristive loop (Figure S7, Supporting Information), and its retention was stable (Figure S8, Supporting Information). A horizontal 2D‐GaO_x_ device with Ag electrodes (Figure S9, Supporting Information) was also developed. However, the *I–V* curve did not indicate threshold or memristive switching, confirming that the distance between the electrodes and the area exposed to air was critical for achieving the desired threshold‐switching behavior. Finally, 2D‐GaO_x_ devices with Ag electrodes of various diameters (Figure S10, Supporting Information) were fabricated; the *I–V* loops all had similar directions, changes in current amplitude, and threshold‐switching windows, indicating that the device performance was independent of this diameter.

Figure 2g reveals that the 2D‐GaO_x_ device achieved a switching speed of 195 ns, significantly faster than that of biological channels (≈40 µs).^[^ ^50^ ^]^ Figure 2h illustrates the diminution of current in relation to interval time, signifying that the device reverted to its high‐resistance state when the bias was removed. The relaxation time was longer for stronger noxious stimuli (Figure 2h), reaching 212 ms for a 6 V stimulus with a 25 ms pulse width. Figure 2i shows the statistical distribution of the threshold voltages for the positive and negative biases of the 50 2D‐GaO_x_ devices. The forward‐bias threshold voltage was 1.6–3 V with a standard deviation (SD), mean, and variance of 0.327, 2.3, and 0.107, respectively. The negative‐bias threshold voltage was −1 to −1.6 V with an SD, a mean, and a variance of 0.145, −1.24, and 0.02, respectively. Moreover, changes in the threshold voltage were documented for 16 devices from the same sample to record the device degradation after storage in air for 0, 6, and 12 months (Figure 2j). After 6 months, the threshold voltage remained stable. Moreover, the nociceptive window decreased slightly (Figure S11, Supporting Information), indicating a minimal reduction in the performance despite long‐term exposure to moist, oxygen‐rich ambient air.

### 2.3 Artificial Nociceptor Mimicked by a 2D‐GaO_x_ Device

**Figure 3:**
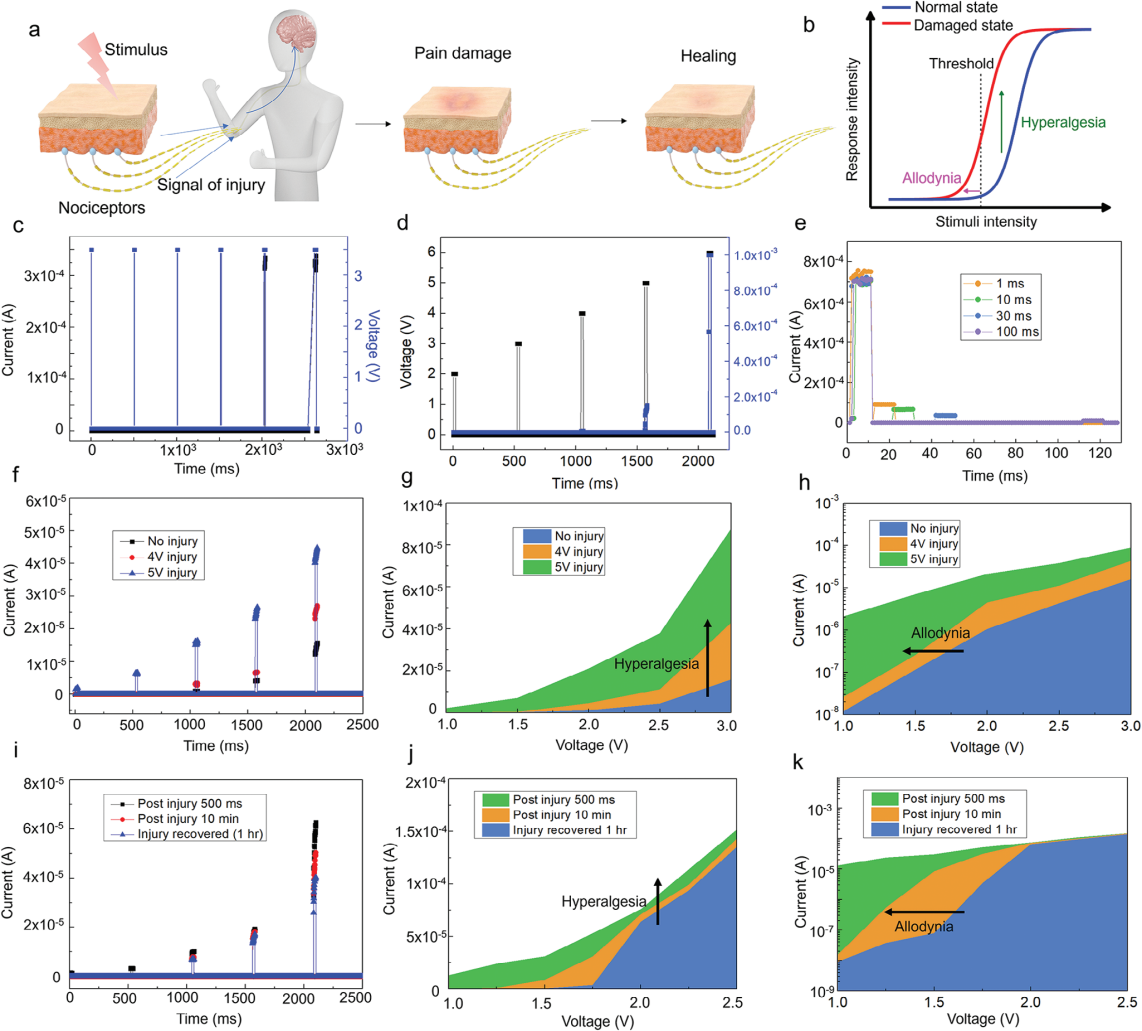
Nociceptive characteristics of the 2D‐GaO_x_ device. a) The physiological safeguard mechanism in human bodies, when subjected to harmful stimuli, encompasses the sensation of pain, recognition of injury, and subsequent healing processes. b) Typical stimulus versus response relationship for normal and damaged nociceptors. c) 3.5 V pulse trains (black) of various pulse widths (1, 3, 5, 10, 15, or 20 ms) and the corresponding output current (blue). d) A train of 20 ms voltage pulses (black) with various amplitudes (2, 3, 4, 5, or 6 V) and the corresponding output current (blue). e) Output currents for 10 ms, 4 V stimuli considering various recovery times. f) Average output currents for pulse amplitudes of 1, 1.5, 2, 2.5, or 3 V applied 1 min after an injury pulse of 0, 4, or 5 V, and these results on g) linear and h) log scales. i) Average output currents for pulse amplitudes of 1, 1.25, 1.5, 1.75, 2, 2.25, or 2.5 V following an injury pulse of 5 V for 25 ms with different recovery times of 500 ms, 10 min, or 1 h, and these results on (j) linear and (k) log scales.

Nociceptors, essentially free nerve endings dispersed across the skin, possess the capability to discern signals emanating from injured tissues.^[^ ^34^ ^]^ They are also known as pain sensors (**Figure** **3**a). A bionic nociceptor must be fast, sensitive, and capable of evaluating stimuli mimicking the biological nervous system and rapidly transmitting signals to the central nervous system through a network.^[^ ^35^ ^]^ Upon exposure to an external stimulus surpassing its threshold, a biological nociceptor initiates an action potential. Consequently, the nociceptor conveys neural signals indicative of a “threat” to the brain, which are interpreted as pain (Figure 3b). In the absence of such stimuli, the nociceptor remains inactive. Nociceptors have four key features: threshold triggering, no adaptation, sensitization, and relaxation, which are discussed for biological and artificial nociceptors below.

In a biological nociceptor, parameters, such as intensity, duration, and number of stimuli, can affect the threshold triggering of the action potential. For the 2D‐GaO_x_ device, when an external stimulus with a given intensity (e.g., 3.5 V) was applied, the device remained inactive until the pulse width reached 10 ms. As the pulse width expanded to 20 ms, the current response increased from 312 to 338 µA (Figure 3c), emulating the biological behavior of stimulus duration and increasing the pain intensity. For all electrical tests, the time interval (500 ms) between pulses was sufficient for the device to relax to its resting state. Device triggering also depends on the number of applied voltage pulses; hence, different training pulses with varying amplitudes (2, 3, 4, 5, and 6 V, with a 20 ms pulse width) were applied (Figure 3d). Similar to biological systems, this study determined the duration and intensity thresholds over which the conductive filaments would bridge the electrodes, transitioning the device from a high‐resistance to a low‐resistance state. The relationship between the stimulus amplitude (4–6 V) and current response with the pulse number (Figure S12, Supporting Information) was investigated. The current through the 2D‐GaO_x_ device gradually increased with the number of stimulus pulses. The interval time between identical stimulus pulses influenced the current output of the 2D‐GaO_x_ device (Figure S13, Supporting Information).

Biological nociceptors do not adapt to continuous noxious stimuli; rather, they continuously send action potentials to protect their bodies. This behavior is called “no adaptation.” As shown in Figure S14 (Supporting Information), the device was activated subsequent to a specific number of pulses, with the number decreasing with increasing pulse amplitude (e.g., 51 pulses at 2.75 V and 1 pulse at 3 V). After the 2D‐GaO_x_ device was switched on, the current response was maintained despite the application of additional voltage pulses, mimicking the biological “no adaptation” behavior.

After a noxious stimulus is removed, the bodily nociceptor signal dissipates; this is known called “relaxation.” First, the output current performance of the device (its effectiveness and responses) was investigated for stimuli of various sizes, durations, and repetitions with long recovery times, which were then shortened to 10–100 ms (Figure 3e). Specifically, a voltage pulse (4 V, 10 ms) was applied to make the device conductive; this was followed by a small reading pulse (1 V, 200 ms) after an interval of 1–100 ms. The current response was then measured (Figure 3e). A shorter time interval corresponded to an elevated reading current, indicating that the device maintained its activity for a brief period subsequent to the removal of the harmful stimulus; complete relaxation required time. The relaxation time increased with stronger noxious stimulus (Figure S15, Supporting Information). This relaxation process is consistent with that of biological nociceptors, which require time to return to their undamaged state after a traumatic injury.

Sensitization involves a reduction in the stimulus threshold (allodynia) and an augmentation of the response (hyperalgesia) of nociceptors after a traumatic nerve injury. This sensitization behavior was also observed in the proposed device. First, the device was subjected to a traumatic injury pulse, characterized by either 4 or 5 V and a pulse width of 5 ms. Subsequently, a sequence of pulse trains with different amplitudes (1, 1.5, 2, 2.5, or 3 V) was applied, and the current response 30 s after the injury pulse ended was recorded. As shown in Figure 3f, different stimuli, such as the 4 and 5 V pulses, caused the device to rapidly transition to a low‐resistance state, augmenting the current for signal transmission. Furthermore, the output current response of the 2D‐GaO_x_ nociceptors was stronger with stronger stimulus. The maximum current outputs for each pulse train in the normal state (blue) and for the 4 and 5 V injury pulses were plotted on logarithmic and linear scales in Figure 3g,h, respectively. After receiving either pulse, the 2D‐GaO_x_ nociceptor entered the injured state, reducing its threshold voltage and facilitating triggering. This results in emulated allodynia. Moreover, for a 2.5 V pulse train, the output current was 240 µA before the injury pulse, increasing to 350 and to 530 µA following the 4 and 5 V injury pulses, respectively; this mimicked hyperalgesia. The stability of the 2D‐GaO_x_ nociceptor was evaluated through cyclic testing, demonstrating its potential reusability for further applications (Figure S16, Supporting Information). Relaxation (i.e., the time after injury) also affects allodynic and hyperalgesic injury responses. To demonstrate this, a 3.5 V injury pulse with a 25 ms width was subjected to the device, followed by a series of pulses of different amplitudes (1, 1.25, 1.5, 1.75, 2, 2.25, and 2.5 V) after various time intervals (500 ms, 10 min, and 1 h). Figure 3i shows the effects of the recovery time on the output current. The current responses shown in Figure 3j,k reveal that a shorter time interval resulted in a lower threshold voltage and higher current, indicating sensitization (allodynia) and an exaggerated response (hyperalgesia) before recovery. Approximately 1 h after a 3.5 V injury pulse, the current intensity was similar to that in the undamaged state, indicating that the device had completely relaxed. Following the application of a 4 V stimulus to the 2D‐GaO_x_ nociceptor, applying −1 or −2 V stimulus caused rapid recovery; the output current of the component decreased rapidly (Figure S17, Supporting Information). This result revealed that the device imitated the recovery of a nociceptor from a traumatic injury over time, and an opposite bias caused recovery from an injured state. The allodynic and hyperalgesic behaviors of the 2D‐GaOx nociceptors did not significantly degrade after 6 months of storage in air (Figure S18, Supporting Information). In summary, the developed Ag/2D‐GaO_x_/SiO_x_/Si nociceptor has a remarkable switching speed of 195 ns, low‐power energy of ≈1.95 × 10^−11^ J, and compliance‐free characteristics. The aforementioned energy value was several orders of magnitude lower than most values reported from other two‐terminal nociceptors (**Table** **1**) previously reported, and its performance was comparable with that of state‐of‐the‐art artificial nociceptors.

**Table 1:** A comparison of the nociceptive behaviors of artificial nociceptors reported in another research. The threshold power was defined using the formula: average threshold voltage (V) × current (A) = power (W). The energy consumption was then computed as power (W) × the switching time (t) = energy (J). The switching speed was defined by the rate at which the current increases to a saturation value after applying the threshold voltage. The recovery time represented the interval required for the current to return to its baseline state. The On/Off ratio was determined by comparing the current levels recorded before and after threshold switching at specific reading voltages. The maximum nociceptive state was defined as the maximum number of read voltages that could achieve different current values after a stimulus injury. The compliance current was defined as the specific current level that prevented the device from undergoing a hard breakdown.

Device structure	On/Off ratio	Switching time	Threshold voltage [V]	Maximum nociceptive state	Short‐term recovery time [s]	Consumption energy [J]	Compliance current [A]	Ref.
Ag/2D‐GaO_x_/SiO_x_/Si	≈10^4^	195 ns	2.3	7	212 ms	1.95 × 10^−11^	N/A	Our work
Ag/SiO_x_:Ag/Pt	10^7^	N/A	0.25	5	10 ms	N/A	10^−5^	[41]
Pt/Ag/SiO_x_‐nanorod/Ag/Pt	10^6^	20 µs	0.78	6	20 ms	1.56 × 10^−10^	10^−4^	[50]
Ag/FK‐800/Pt	10^3^	340 µs	3.5	5	N/A	1.19 × 10^−7^	10^−5^	[61]
Al/POT2T/MAPbBr_3_/NiO_x_/PEDOT:PSS/ITO	10^5^	60 µs	≈4	4	≥20 ms	2.4 × 10^−7^	10^−3^	[62]
Ti/Au/MoS_2_/Ag/Au	10^6^	N/A	0.21–0.26	5	10 ms	N/A	10^−4^	[63]
Ag/FF MR/Ag	10^5^	400 µs	1.64	5	100 ms	≈9.6 × 10^−7^	2.5 × 10^−5^	[64]
Ag/CιC/ITO/PET	>10^4^	N/A	0.4	5	10 min	N/A	10^−4^	[65]

### 2.4 Elemental Characterization of the 2D‐GaO_x_ Layer After Threshold Switching

**Figure 4:**
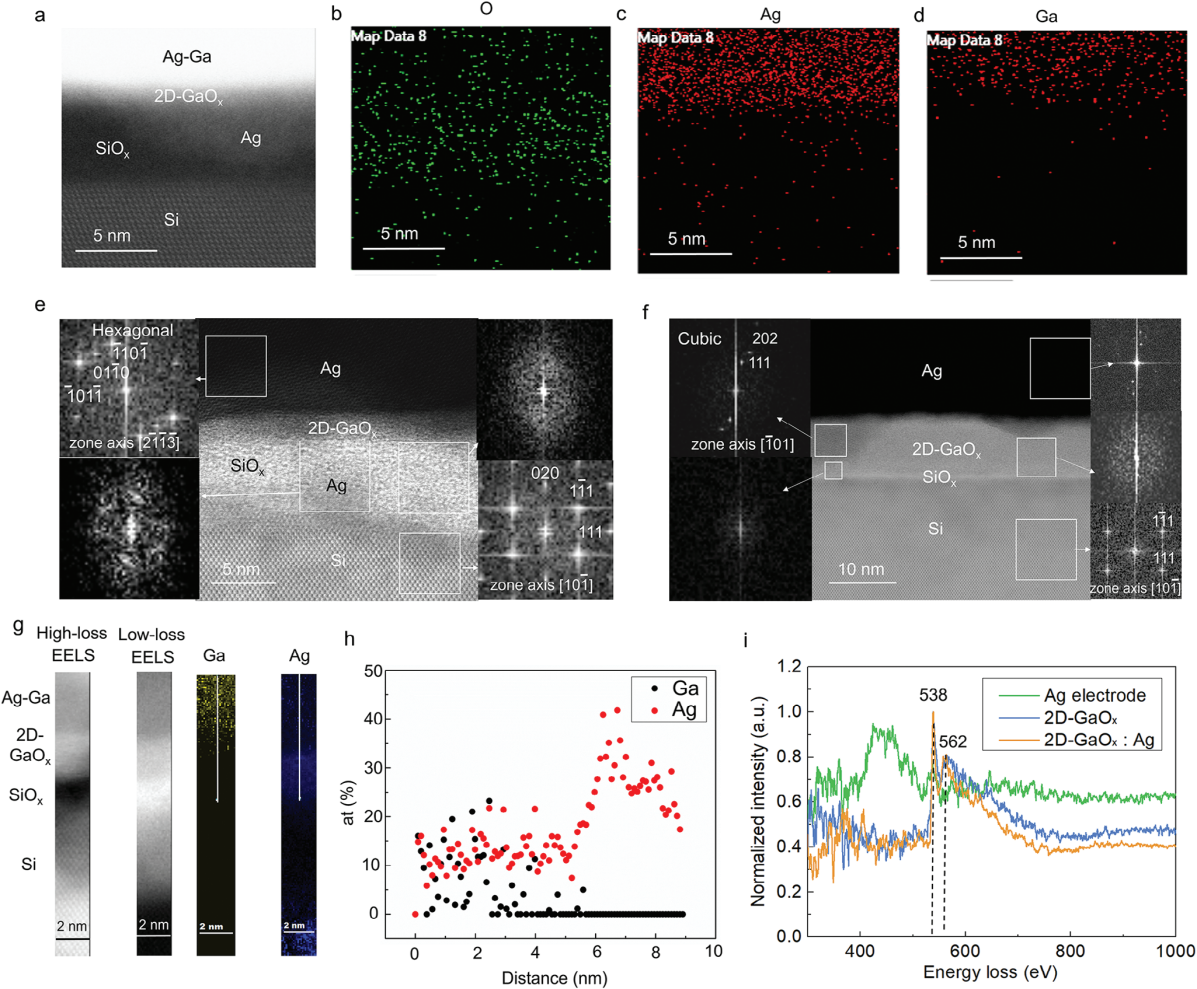
TEM evidence of Ag filament formation in the 2D‐GaO_x_ device after threshold switching. a) Cross‐sectional STEM‐HAADF image of a 2D‐GaO_x_ device after a 6 V electrical stimulus. b–d) STEM‐EDS mapping of elemental O, Ag, and Ga, respectively, from (a). e,f) HRTEM images of the Ag/2D‐GaO_x_/SiO_x_/Si structure after threshold switching for 2D‐GaO_x_ layers of various thicknesses. g) STEM–EELS high‐loss, low‐loss, elemental Ga, and elemental Ag mapping of a 2D‐GaO_x_ device after a 6 V electrical stimulus. h) EELS element line‐scan for the cross‐sectional white lines in the Ga and Ag mapping images in (g). i) EELS high‐loss spectra captured from the Ag electrode (green), 2D‐GaO_x_ (blue), and 2D‐GaO_x_:Ag (orange).

Scanning transmission electron microscopy (STEM) was employed to analyze the component materials and verify the device structure. STEM energy‐dispersive X‐ray spectroscopy (EDS) elemental analysis was performed to verify the presence of dispersed Ag in the 2D‐GaO_x_ nociceptors after threshold switching (**Figure** **4**a–d). Ag atoms can easily intermix with 2D‐GaO_x_, making it challenging to identify the interface between the 2D‐GaO_x_ and Ag (Figure 4a). Figure 4b–d shows elemental O, Ga, and Ag EDS mappings selected from the dark‐field STEM image. EDS mappings revealed that the elemental Ag dots migrated from the Ag electrode in response to the electromotive force. To investigate the mechanisms by which Ag was dispersed and conducted in the amorphous SiO_x_ matrix following electrical stress, a thick SiO_x_ device without the 2D‐GaO_x_ film was fabricated, and electrical stress was applied (Figure S19, Supporting Information). Many Ag clusters can easily diffuse into the SiO_x_ matrix, contaminating the heavily doped Si substrate without allowing recovery and causing the device to transition to a low‐resistance state. A previous first‐principles study calculated the barrier to the migration of single Ag atoms into amorphous SiO_x_ through bulk diffusion and demonstrated that Ag cations can easily diffuse interstitially through SiO_x_.^[^ ^51^ ^]^ Therefore, depositing the 2D‐GaO_x_ film on the SiO_x_ layer enables easy accommodation of the Ag filaments, resulting in a device transition with substantial bias.

The relationship between the active switching area and thickness of the 2D‐GaO_x_ layer, which should be the area of the GaO_x_‐SiO_x_ matrix, was investigated by performing TEM on 2D‐GaO_x_ devices with 2D‐GaO_x_ layers of various thicknesses (Figure 4e,f). Figure 4e shows the diffraction patterns inside the GaO_x_‐SiO_x_ matrix and Ag electrode following a 6 V stimulus, confirming the presence of elemental Ag. The diffraction patterns of the Ag electrode and GaO_x_‐SiO_x_ indicated that the Ag phases in the electrode and matrix were hexagonal and cubic, respectively. After the electrical stimulus was removed, the Ag filaments in the 2D‐GaO_x_ layer were confirmed to have ruptured. After consecutively applying 6 V stimuli, many Ag clusters regrouped in the 2D‐GaO_x_ layer, increasing the current of the 2D‐GaO_x_ device (Figure S20, Supporting Information). Moreover, after a 12 V stimulus, many Ag clusters easily reached the Si electrode without recovery, resulting in a hard breakdown of the 2D‐GaO_x_ device (Figure S21, Supporting Information).

To better understand the Ag electron configurations in the GaO_x_‐SiO_x_ matrix, a STEM–EELS investigation was performed on cross‐sections of the 2D‐GaO_x_ nociceptor after preparing it using a focused ion beam. Figure 4g shows the STEM–EELS spectra and elemental maps of the 2D‐GaO_x_ device following an electrical stimulus. The figure revealed the differences in the electronic configurations of each layer and confirmed that Ag diffusion occurred after the stimulus. Figure 4h compares the EELS spectra of the Ag electrode, 2D‐GaO_x_:Ag, and 2D‐GaO_x_. To facilitate comparison, all spectra were normalized, and the background intensities were removed. The O‐*K* edges of the 2D‐GaO_x_ and 2D‐GaO_x_:Ag spectra were similar; both had O‐*K* edges at 538, 562, and 565 eV, indicating oxygen orbital peaks.^[^ ^52^, ^53^ ^]^ Moreover, the EELS measurements revealed Ag peaks in the 2D‐GaO_x_:Ag spectrum, confirming that Ag diffused into the 2D‐GaO_x_ layer after applying a stimulus exceeding the threshold. Furthermore, the elemental Ga remaining from the liquid metal printing process diffused into the GaO_x_‐SiO_x_ matrix. The main Ag spectra were difficult to measure using EELS because the Ag‐*N* edges were at 56 and 63 eV; thus, these edges were easily covered or disturbed by the Ga‐*M*_4,5_ edges (Figure S22, Supporting Information). Therefore, the Ag‐*M*_4,5_ edges near 360–420 eV were measured to confirm the electronic structure and reconstruction of Ag in the 2D‐GaO_x_ layer (Figure 4i). These Ag‐*M*_4,5_ edges have rarely been reported in the literature,^[^ ^54^ ^]^ and no high‐energy‐resolution references regarding Ag were found. The spectra exhibited several pre‐edges at ≈360–420 eV, which were primarily attributed to transitions from the 3*d* state to *p* final states.^[^ ^55^ ^]^ Electronic reconstruction occurred in 2D‐GaO_x_:Ag; the charge redistribution between orbitals (Ag‐*p* → Ag‐*s*,*d*) was consistent with the observed charge redistribution in the Ag edges of the Ag electrode ranging from 300 to 420 eV.^[^ ^56^ ^]^ Some differences were observed at the Ag‐*M*_4_ and Ag‐*M*_5_ edges of the 2D‐GaO_x_:Ag spectrum at 323 and 363 eV, respectively, where many *p*‐type states of Ag were in the unoccupied density of states, indicating that the valence state of Ag in the 2D‐GaO_x_:Ag EELS spectrum was lower than that of Ag in the Ag‐electrode EELS spectrum.^[^ ^57^ ^]^ Unfortunately, transitions from the 3*d* states to the *p* states produced weak peaks.^[^ ^56^ ^]^ Thus, precise charge transfer at the Ag orbitals could not be confirmed using EELS because the resolved Ag spectra had poor signal‐to‐noise ratios. However, these results confirmed that Ag atoms diffused into the oxide layer with charge transformation into Ag cations after a threshold electrical stimulus, and the observed charge transfer occurred owing to the formation of Ag─O bonds in the 2D‐GaO_x_ layer.

The HRTEM images and STEM–EELS analysis suggested that Ag clusters were easily generated from the Ag electrode under a threshold electrical stress. Therefore, Ag clusters were dispersed in the GaO_x_‐SiO_x_ matrix after threshold switching. The Ag electrode generated conductive filaments in the 2D‐GaO_x_ device, facilitating the creation of a conduction pathway and allowing the 2D‐GaO_x_ device to transition to a low‐resistance state. Filaments bridging the Ag electrode and substrate formed when a threshold voltage pulse of sufficient duration was applied, and the 2D‐GaO_x_ device switched from a high‐resistance to a low‐resistance state. The decrease in current after the elimination of the electrical stress was ascribed to the rupture of the conductive Ag filaments, which minimized the interfacial energy.

### 2.5 Conduction Mechanism of the 2D‐GaO_x_ Nociceptor

**Figure 5:**
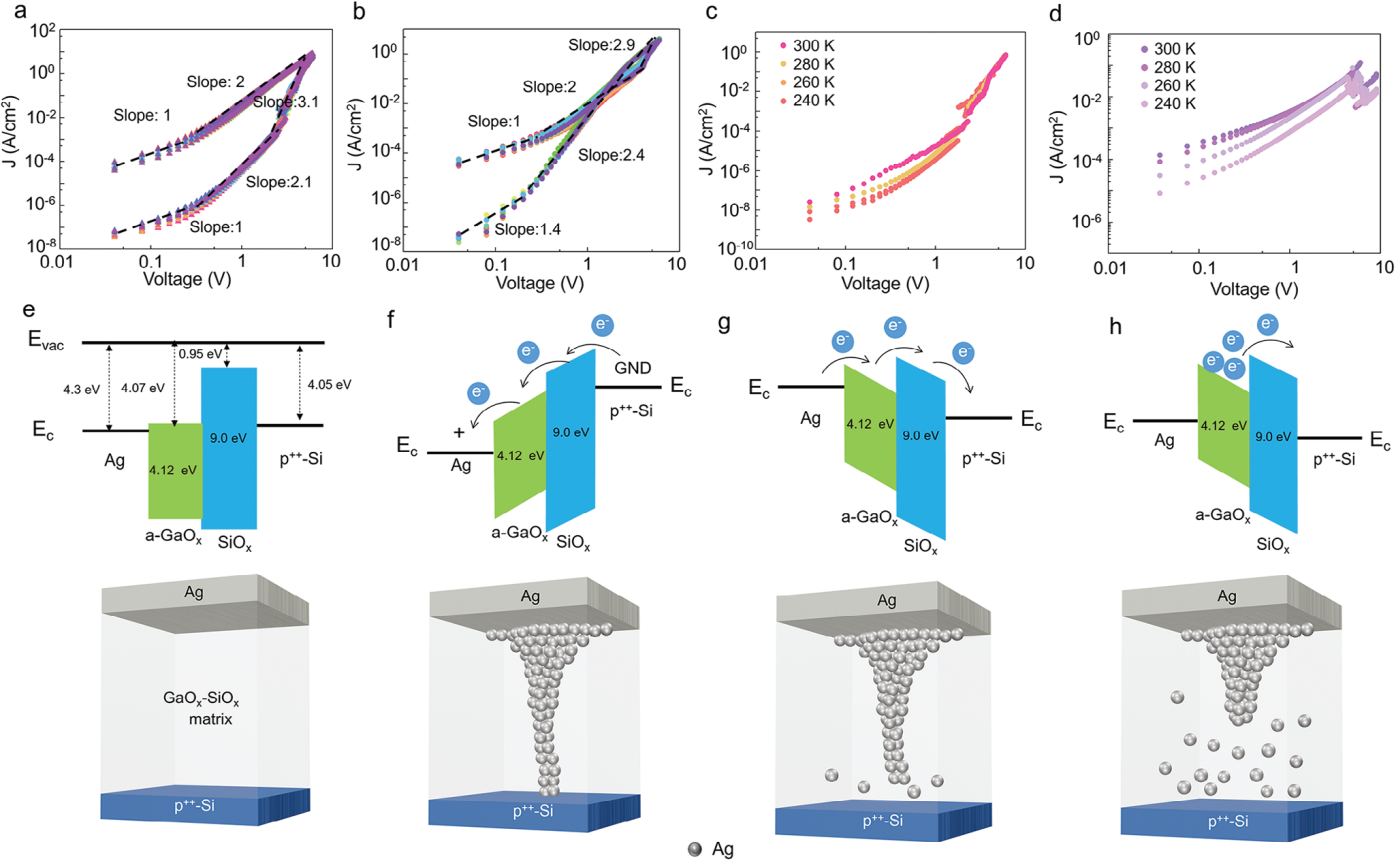
Charge transport mechanism and illustration of Ag filament conduction in the 2D‐GaO_x_ nociceptor. a,b) Conduction mechanism fitting curves for positive and negative sweeps, respectively, for the SCLC conduction mechanism. Ten sweeping cycles were obtained. c,d) Temperature‐dependent sweeping performance for the high‐ and low‐resistance states, respectively, at temperatures ranging from 240 to 300 K. e–h) Illustration and corresponding band diagrams of the formation and rupture of Ag filaments in the 2D‐GaO_x_ nociceptor.

The *I–V* sweeping performance was examined to study the charge transport behavior of the 2D‐GaO_x_ devices. Because the current response was symmetrical, the currents for a positive bias were fitted with possible charge transport equations at different stages after dividing these stages by the threshold voltage. Testing with various charge transport models suggested that the charge conduction across the metal–insulator–semiconductor junctions was most consistent with the space‐charge‐limited current (SCLC) model, as indicated by the log *J* versus log *V* curves in **Figure** **5**a,b. Based on their slopes, the curves can be divided into three charge conduction regimes. In a low‐bias domain, denoted as Region 1, there was a linear correlation between current density and voltage (the slope of the log *J* vs log *V* curve was close to 1), implying ohmic conduction. In this region, the thermally generated free carriers present in the material generated a current, expressed as follows:^[^ ^58^ ^]^

(1) $$J=en_{0}\mu \frac{V}{d}$$

where *J* is the current density, *e* is the charge of an electron, *n*_0_ is the free‐electron density in thermal equilibrium, *µ* is the carrier mobility, and *d* is the film thickness. Increasing the voltage‐enhanced charge injection. At the transition voltage, the slope changed to 2, a defining characteristic of the SCLC model. This regime is termed as Region 2, within which the introduced carriers filled the traps located in the prohibited zone. For an isolated singular shallow trap, the current density adheres to the trap‐limited formula in the SCLC model:^[^ ^59^ ^]^

(2) $$J=\frac{9\mu \theta \varepsilon_{r}\varepsilon_{0}V}{8d^{3}}$$

where *θ* is the ratio of free to trapped carriers, ε_0_ is the vacuum permittivity, ε_r_ is the optical dielectric constant, and *d* is the film thickness. Further increasing the bias strengthened the carrier injection, leading to a gradual filling of the traps. All traps were filled at the trap‐filled voltage limit. Consequently, there was an abrupt incline in the slope from 2, leading to a sharp surge in current density as the introduced carriers transitioned directly into the conduction band. This regime is denoted as Region 3. For voltage biases in this region, a space charge was established at the interface, thus constraining additional charge carrier injection from the electrode. Thus, the current density began to exhibit a trap‐free behavior with a slope of 2. This behavior is known as trap‐free Child's law. Accordingly, the plot of log *J* versus log *V* should be temperature‐dependent.^[^ ^60^ ^]^ The current response increased with the temperature, implying that the charge transport mechanism in the 2D‐GaO_x_ devices was temperature‐related (Figure 5c). Moreover, this suggested that charge‐trapping and charge‐detrapping processes, which can occur owing to the many trap states induced by spontaneous Ag ion migration, may also cause threshold switching. Generally, the switching mechanism of the 2D‐GaO_x_ device can be denoted as a dynamic, non‐equilibrium charge‐trapping process. When a high bias voltage was given to the electrode, it caused the trap level to fall below the Fermi energy level, leading to the subsequent electron injection from the Ag electrode into the trap sites. Charge transport in the low‐resistance state can be explained by an effective detrapping process that presumably begins at the Ag–Si interface after the Ag filament connection. The *I–V* slope was altered from that of the trapped‐filled limit region to that of the ohmic region and was temperature‐dependent (Figure 5d).

Based on these results, an Ag‐based threshold‐switching mechanism for the 2D‐GaO_x_ nociceptor is proposed. Figure 5e–h presents the conduction mechanism and the corresponding band diagram for Ag clusters in the 2D‐GaO_x_‐SiO_x_ matrix. No Ag clusters were formed in the GaO_x_‐SiO_x_ in the initial state shown in Figure 5e (before the electrical stimulus). Ag electromigration was initiated from the top Ag electrode when the Ag electrode was positively biased and the heavily doped Si substrate was grounded. As shown in Figure 5f, once the nociceptor was triggered with an above‐threshold stimulus of 6 V, the Ag cations locally formed a high‐current path in the 2D‐GaO_x_. A previous first‐principles study calculated the barriers to the migration of a single Ag atom in amorphous SiO_x_ through bulk diffusion.^[^ ^51^ ^]^ The results revealed that Ag cations interstitially diffused through amorphous SiO_x_. Under the action of an external electric field, Ag cations moved to the negative end and obtained electrons, resulting in the accumulation of Ag atoms and the formation of conductive filaments. When moving toward the negative end, some Ag ions are attached to the Ag electrode. Thus, applying an external bias above the threshold voltage caused conductive Ag filaments to form through the electromigration of the Ag atoms connecting the two electrodes, leading to the observed switching from a high‐ to a low‐resistance state. Once the electrical stimulus was removed, the conductive Ag filaments spontaneously broke owing to the interstitial diffusion of Ag driven by the need to minimize the surface energy (Figure 5g). The accumulated electrons trapped under the applied electric field can also cause spontaneous charge detrapping, causing the device to return to its initial state (Figure 5h). When a threshold voltage pulse was reapplied to the device, the conductive filament regrouped from the thinnest region, enabling electrical switching from a high‐resistance to a low‐resistance state. Oxygen vacancy migration may have also been involved in the switching process of the 2D‐GaO_x_ device; however, it was not considered to be the primary mechanism because vacancy migration reactions were weaker than those of Ag cation migration.

## 3 Conclusion

This study clarified the conduction mechanism and threshold‐switching behavior of 2D‐GaO_x_ artificial nociceptors fabricated through liquid metal printing. Control and threshold switching between different states of nociceptive function were primarily attributed to the formation of Ag filaments. When the applied external bias exceeded a specific threshold, Ag cations in the oxide matrix of the 2D‐GaO_x_ nociceptor accumulated, causing the conductance to vary in proportion to the total charge transferred. The Ag ion dynamics of the device resembled those of biological nociceptors, and the device exhibited allodynic and hyperalgesic behaviors. Moreover, the 2D‐GaO_x_ layer is vital for regulating and altering the diverse capacities of the devices (from memristor‐like to nociceptor‐like). Therefore, employing heterostructural engineering for fabricating 2D‐GaO_x_ devices is a significant approach for developing energy‐efficient and fast‐switching nociceptors. The devices developed in this study can be miniaturized, representing a pivotal advancement in the development of artificial nociceptors based on 2D materials.

## 4 Experimental Section

### Printing Large‐Area 2D‐GaO_x_ Films

Si (100) was adopted as the substrate, and the native oxide was removed using hydrofluoric acid etchant. A 1–5‐nm‐thick layer of SiO_x_ was deposited through plasma‐enhanced chemical vapor deposition in a process environment of 80 °C containing SiH_4_ and N_2_O as precursor gases. To print 2D‐GaO_x_ on the SiO_x_/Si substrate, the substrate, copper foil, and liquid Ga were first preheated at 40 °C for 30 min on a glass slide. Copper foil was then employed to eliminate the surface oxide layer of residual Ga and was applied with pressure to the preheated Ga for 5–10 s to generate a fresh 2D‐GaO_x_ layer. Following the transfer of the 2D‐GaO_x_ onto the SiO_x_/Si substrate, Kapton tape was used to eliminate residual Ga. The Kapton tape was pressed onto the 2D‐GaO_x_ film to remove residual Ga. Subsequently, a cotton swab soaked in alcohol was used for cleaning at 60 °C. Immediately after cleaning, the dry end of the swab was employed to remove the alcohol, preventing any watermarks.

### Characterization

TEM (JEM‐ARM200F) and cold field‐emission SEM (Hitachi‐SU8010) were used to examine the crystal structure and surface morphology of the 2D‐GaO_x_, respectively. XPS spectra were obtained employing a PHI Quantera II apparatus, utilizing monochromatic Kα X‐ray emissions. PL measurements were conducted with a ×40 objective (having a numerical aperture of 0.6) employing a 266 nm excitation light source, all at ambient temperature.

### Device Fabrication

Standard photolithography and lift‐off processes were used to apply the top Ag electrode. The 60‐nm‐thick Ag top electrode was then evaporated with an evaporation rate of 0.2 nm s^−1^, which was followed by a lift‐off process. After the lift‐off process, the device was annealed at 200 °C for 30 min to eliminate residue contamination.

### Electrical Properties

The current–voltage characteristics and nociceptive data of the 2D‐GaO_x_ devices were measured using a KeySight B1500‐A semiconductor parameter analyzer in a dark box at room temperature. While a DC voltage was applied to the Ag electrode, the p^++^‐Si substrate was grounded.

## Conflict of Interest

The authors declare no conflict of interest.

## Supporting information

Supporting Information

## Data Availability

The data that support the findings of this study are available in the supplementary material of this article.
