# Supplementary material for: Embedded Hybrid‐Dimensional Heterointerface for Filament Modulation in 2D Material‐Based Artificial Nociceptor
Source: Adv Sci (Weinh). 2024 Aug 5;11(36):2401946. doi: 10.1002/advs.202401946 (PMC11422813; doi:10.1002/advs.202401946)
Supplement: Supplementary file 1 — Supporting Information [file ADVS-11-2401946-s001.docx]

**Supporting Information**

Embedded Two-/Three-Dimensional Heterointerface for Filament Modulation in 2D-Based Artificial Nociceptor

Chang-Hsun Huang,^1^ Te-Yu Cheng,^2^ Chia-Yi Wu,^1^ Kuan-Hung Chen,^1^ Tian-Li Wu,^3^ Yi-Chia Chou^*,1^

- 1. Department of Materials Science and Engineering, National Taiwan University, Taipei, Taiwan
  2. Department of Physics, National Yang Ming Chiao Tung University, Hsinchu, Taiwan
  3. International College of Semiconductor Technology, National Yang Ming Chiao Tung University, Hsinchu, Taiwan

*Email: ycchou@ntu.edu.tw


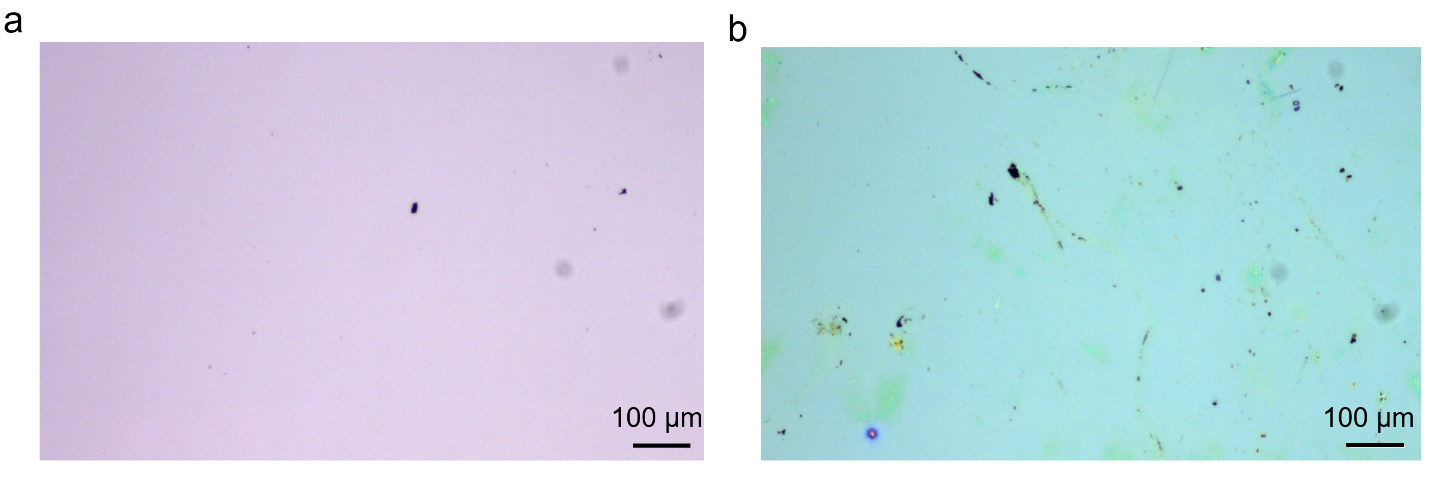


**Figure S1.** The morphology of Si substrate after liquid metal printing of Ga metal. (a, b) Optical images of Si substrate before and after liquid metal printing of Ga metal, respectively.


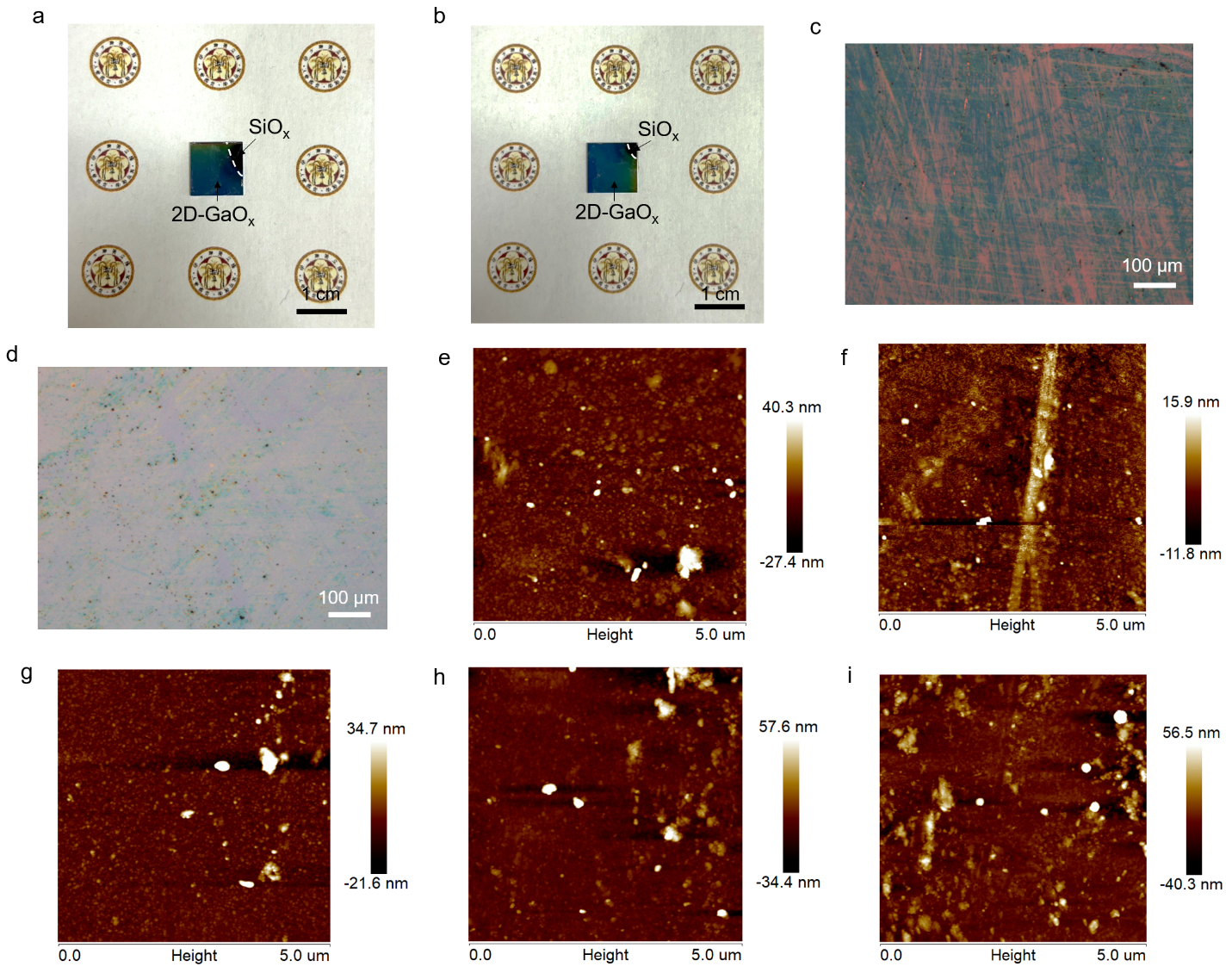


**Figure S2**. Sample images of 2D-GaO_x_ on the SiO_x_/Si substrate after removing residual Ga. (a, b) Images of two samples of 2D-GaO_x_, each exceeding dimensions of 0.5 cm x 0.5 cm on the SiO_x_/Si substrate. (c, d) Optical images of 2D-GaO_x_ adapted from (a) and (b), respectively. (e-i) AFM images of a 2D-GaO_x_ sample adapted from (b). The RMS values of the AFM images are 4.29, 7.37, 2.68, 5.17, and 3.24, respectively.


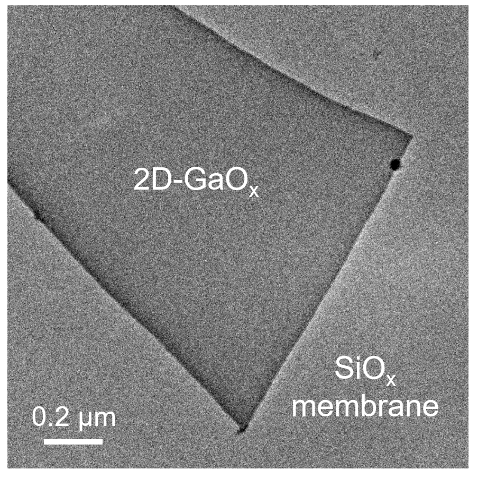


**Figure** **S3.** Low-magnification TEM image of 2D-GaO_x_ printed on TEM SiO_x_ membrane. The black dots represent residual Ga.


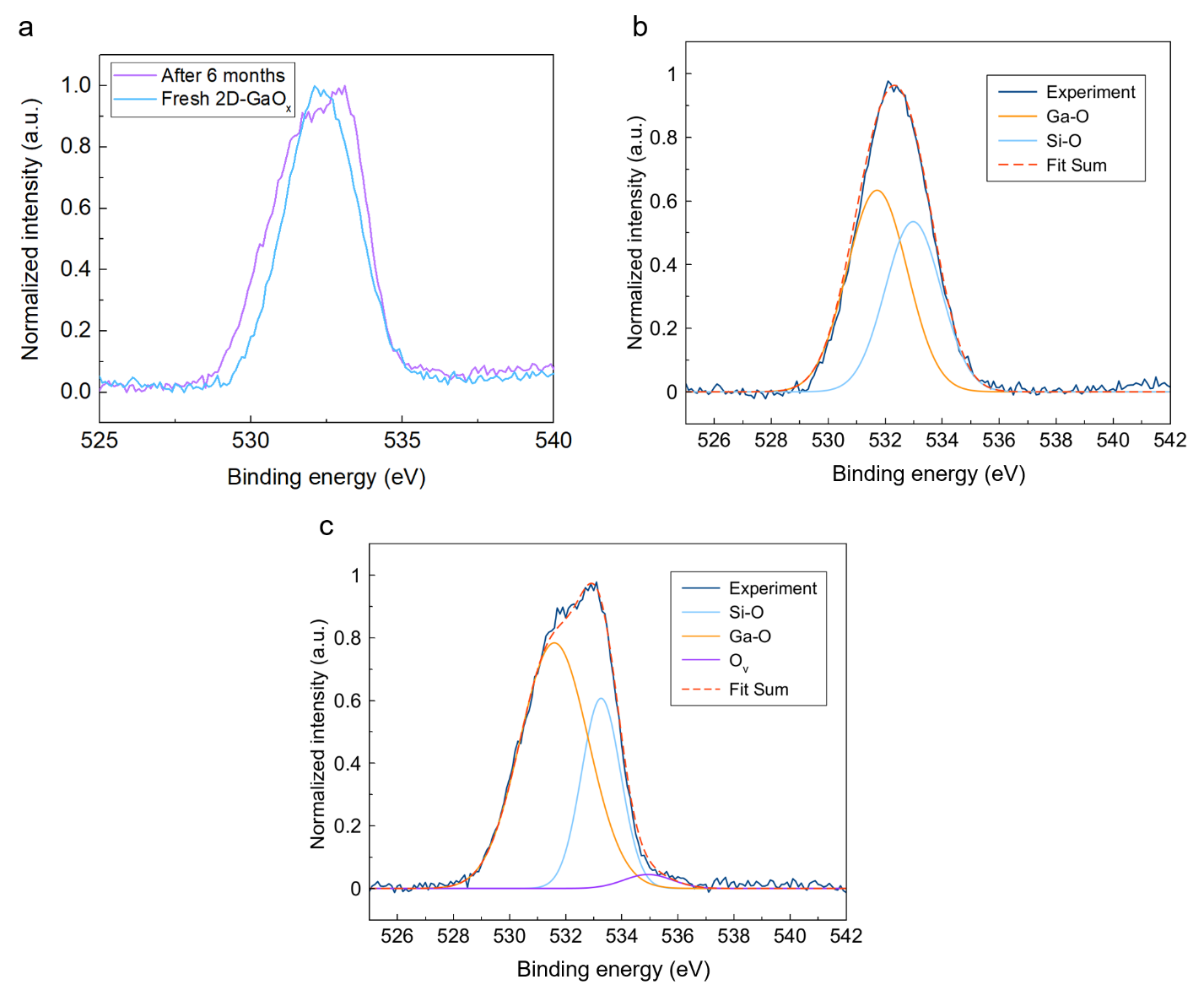


**Figure** **S4.** O1s XPS spectrum of 2D-GaO_x_ printed on SiO_x_/Si substrate. (a) XPS spectrum of fresh 2D-GaO_x_ film and its film after six months stored in air atmosphere. (b, c) Deconvoluted XPS adapted from (a), respectively.


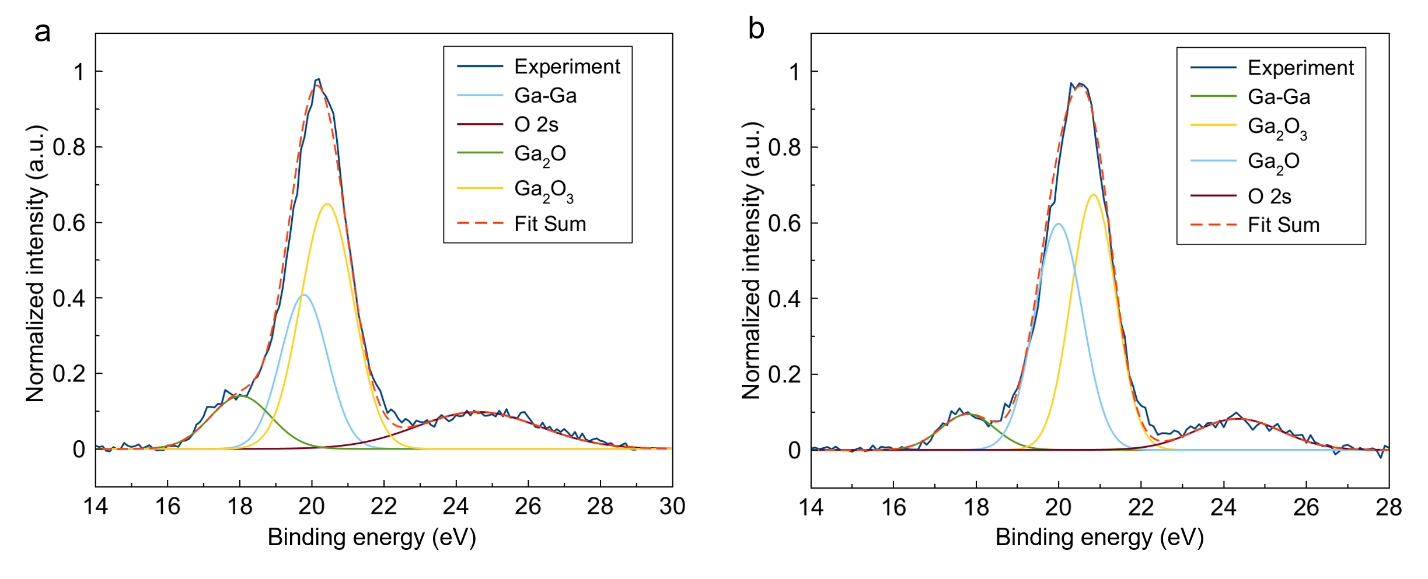


**Figure** **S5.** Ga3d XPS spectrum of 2D-GaO_x_ printed on SiO_x_/Si substrate. (a, b) Deconvoluted XPS spectra of fresh 2D-GaO_x_ film and its film after six months stored in air atmosphere, respectively.


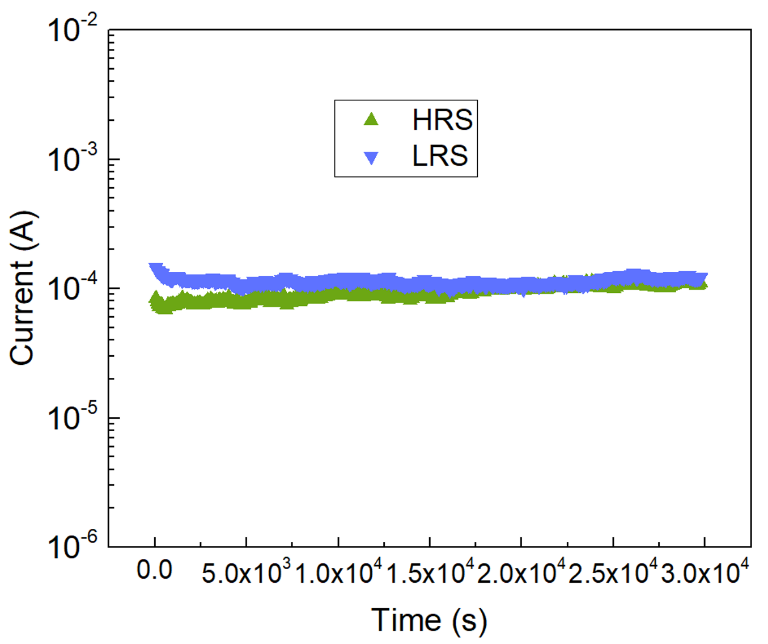


**Figure** **S6.** Retention test of a vertical device with Ag/2D-GaO_x_/SiO_x_/Si structure. The read voltage is set as 0.5 V.


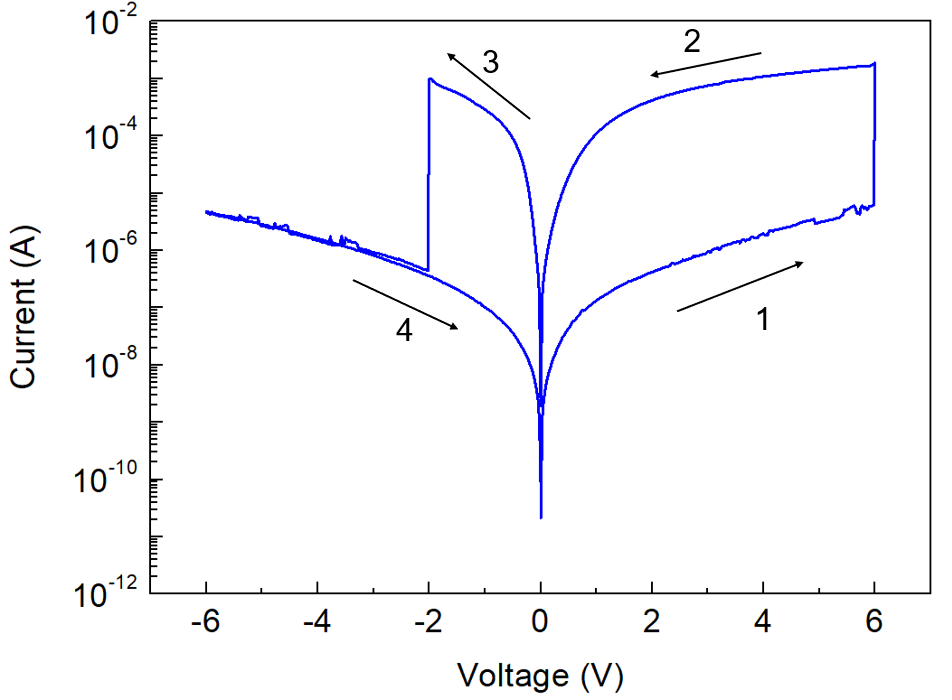


**Figure** **S7.** *I-V* curve of a horizontal 2D-GaO_x_ device with Ag electrode. The loop directions are marked with black arrows.


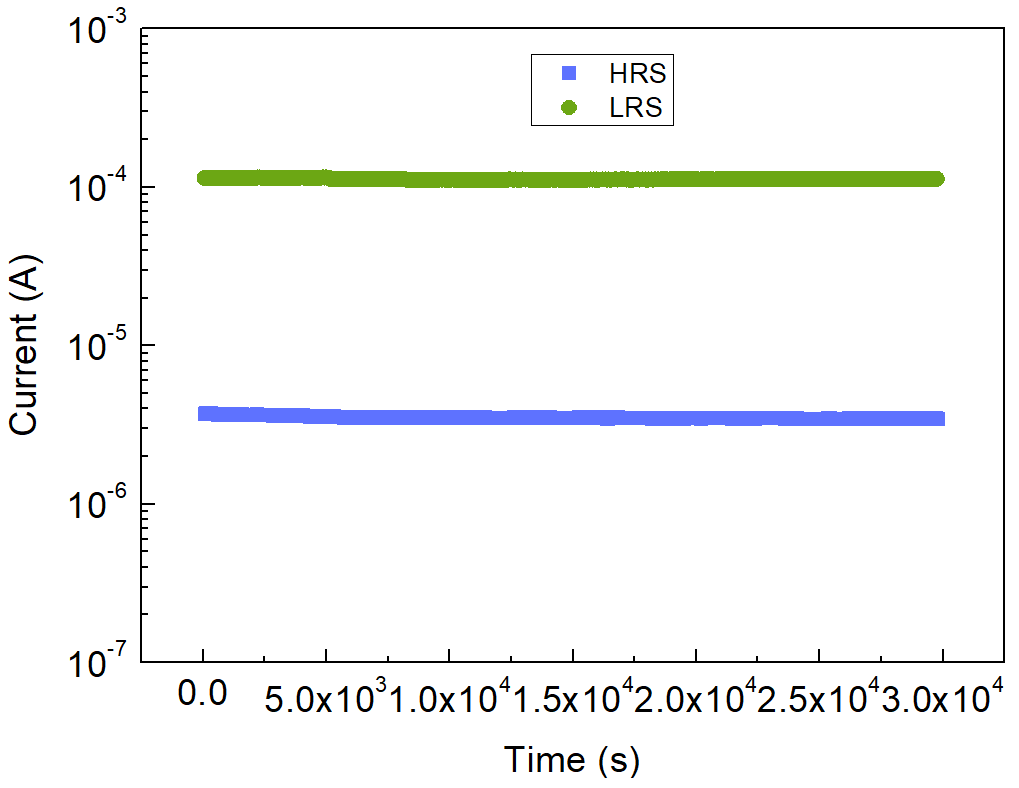


**Figure** **S8.** Retention test of a vertical device with Ag/SiO_x_/Si structure. The read voltage is set as 0.5 V.

**
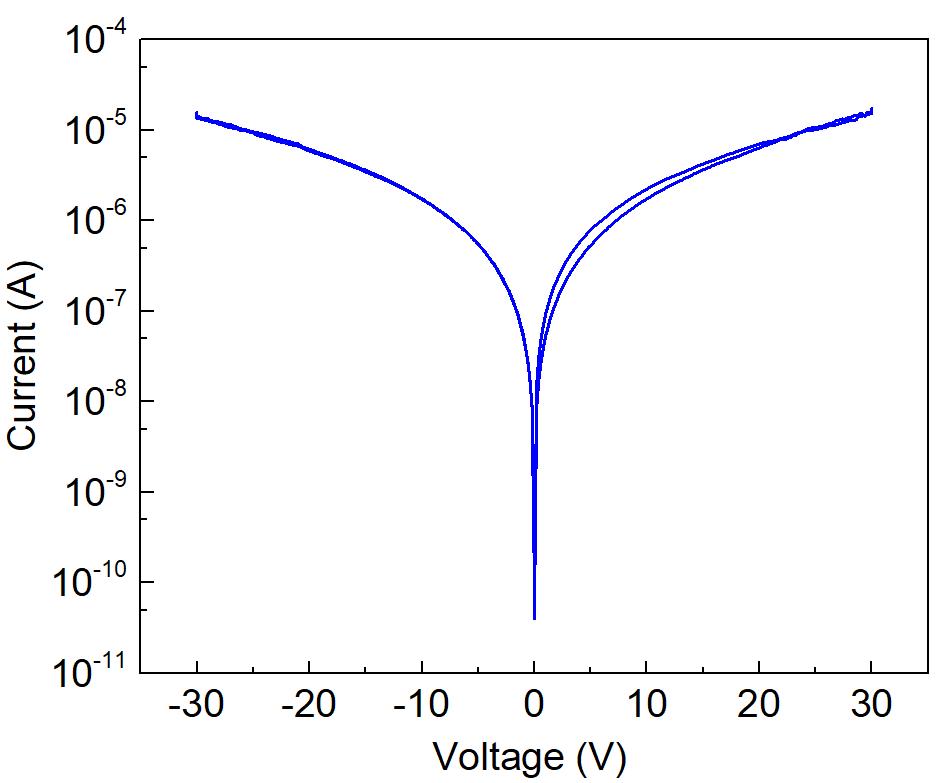
**

**Figure S9.** *I-V* curve of a horizontal 2D-GaO_x_ device with Ag electrodes. The spacing of the electrodes is set as 2 μm.

**
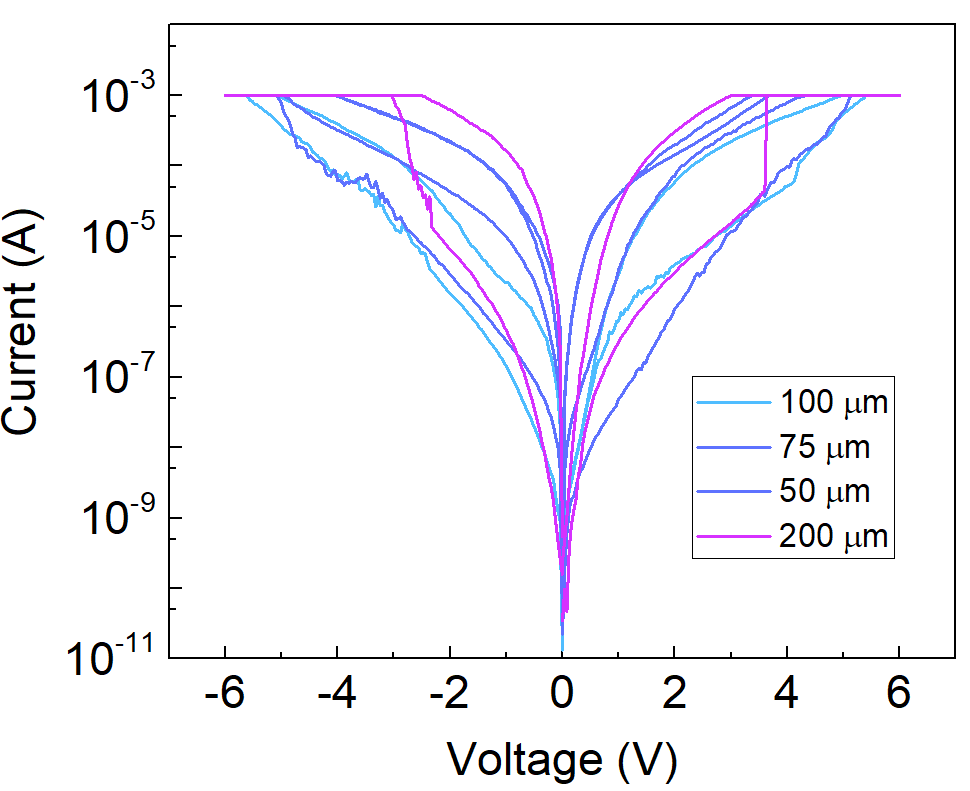
**

**Figure S10.** *I-V* curves of 2D-GaO_x_ devices with Ag electrode diameter ranging from 50 μm to 200 μm.


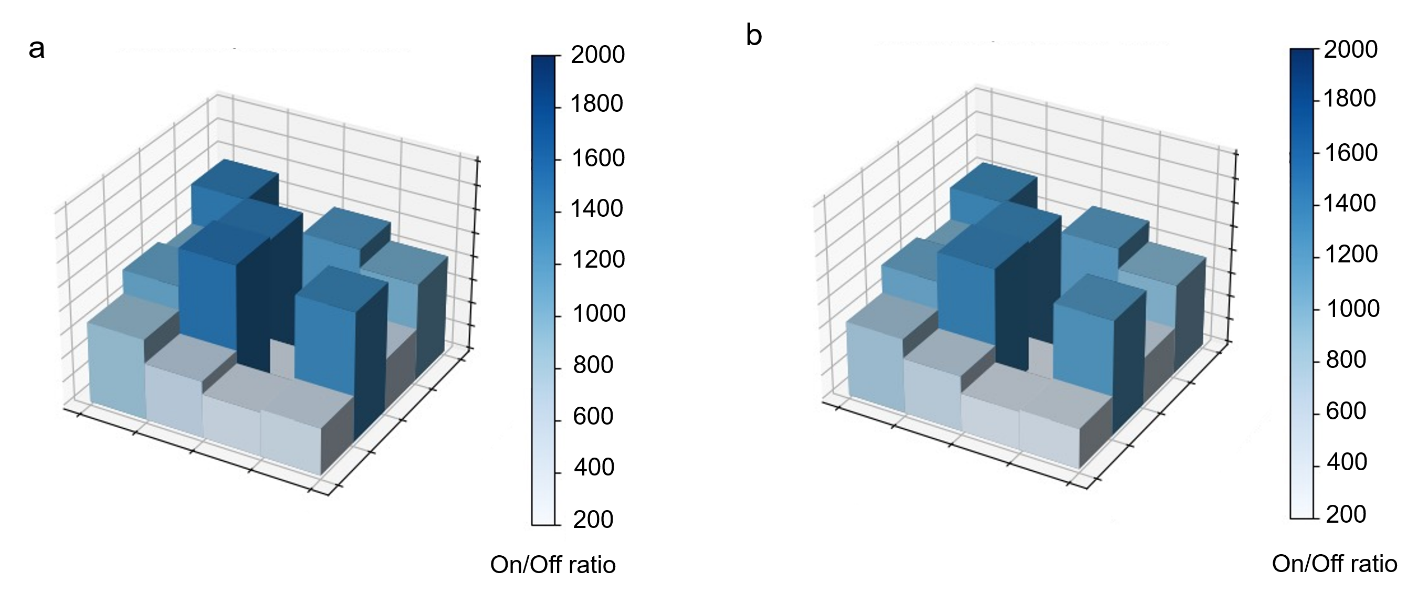


**Figure S11.** The recorded nociceptive window of 16 2D-GaO_x_ devices (a) before and (b) after 6-month storage at air atmosphere.


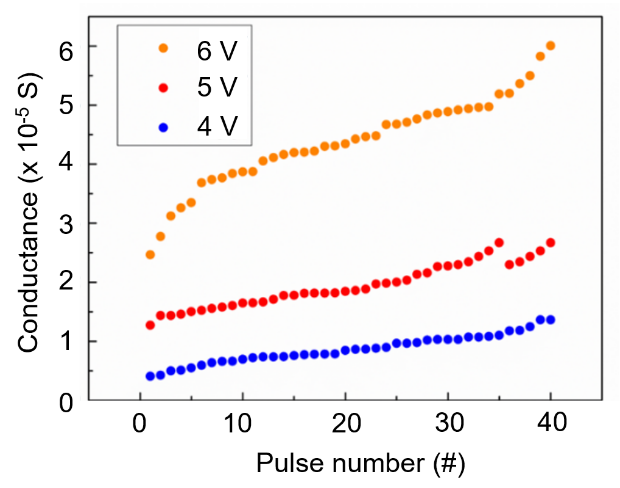


**Figure S12.** Conductance versus pulse number of a 2D-GaO_x_ nociceptor with the input voltage bias varied from 4 to 6V.


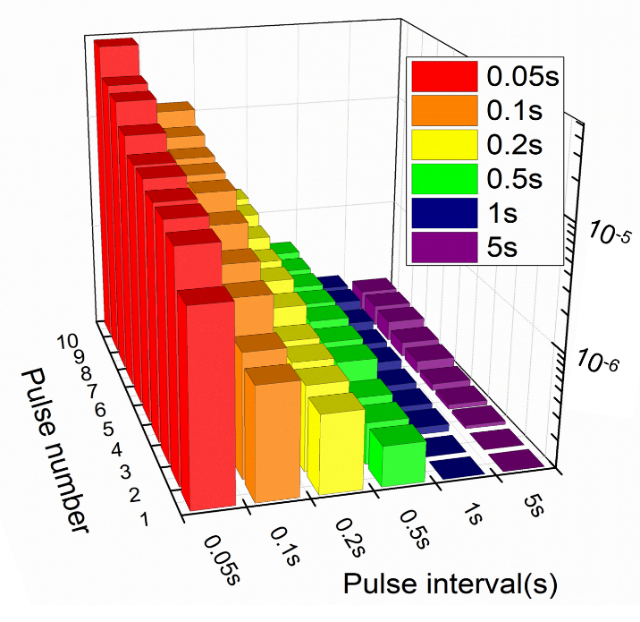


**Figure S13.**  Current response of a 2D-GaO_x_ nociceptor utilizing 6 V pulses with different pulse intervals varied from 0.05 s to 5 s. The device is read at 0.3 V after each pulse.


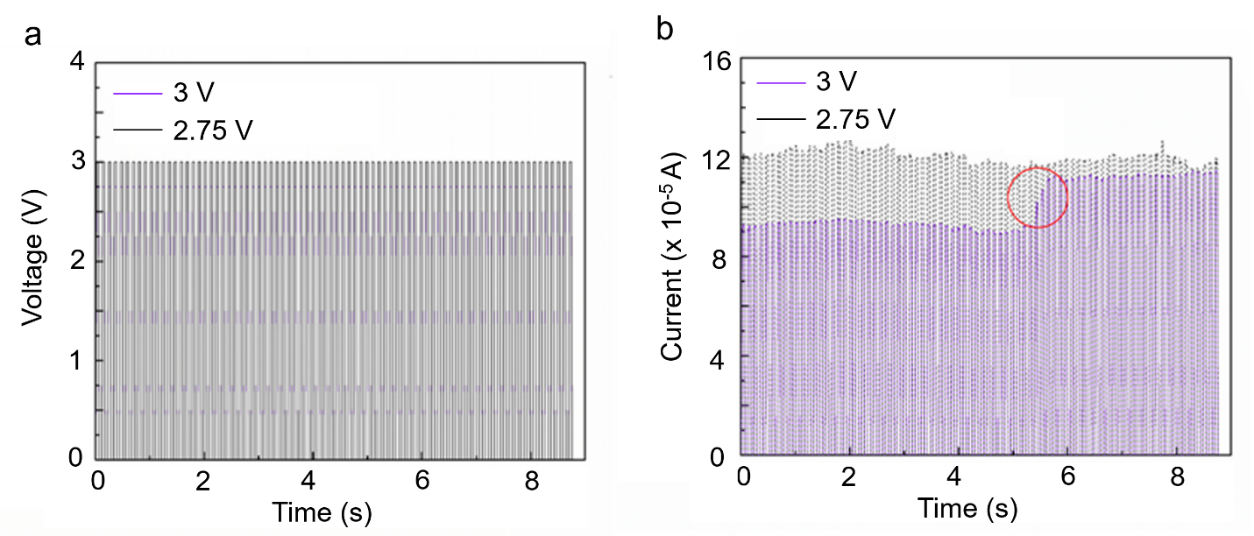


**Figure S14.** A 2D-GaO_x_ nociceptor with no adaptation characteristic. (a) 80 voltage pulses (pulse width of 5 ms and pulse interval of 5ms) applied to a 2D-GaO_x_ nociceptor. (b) Current response of the device to 80 voltage pulses with different amplitude (2.75 and 3 V).


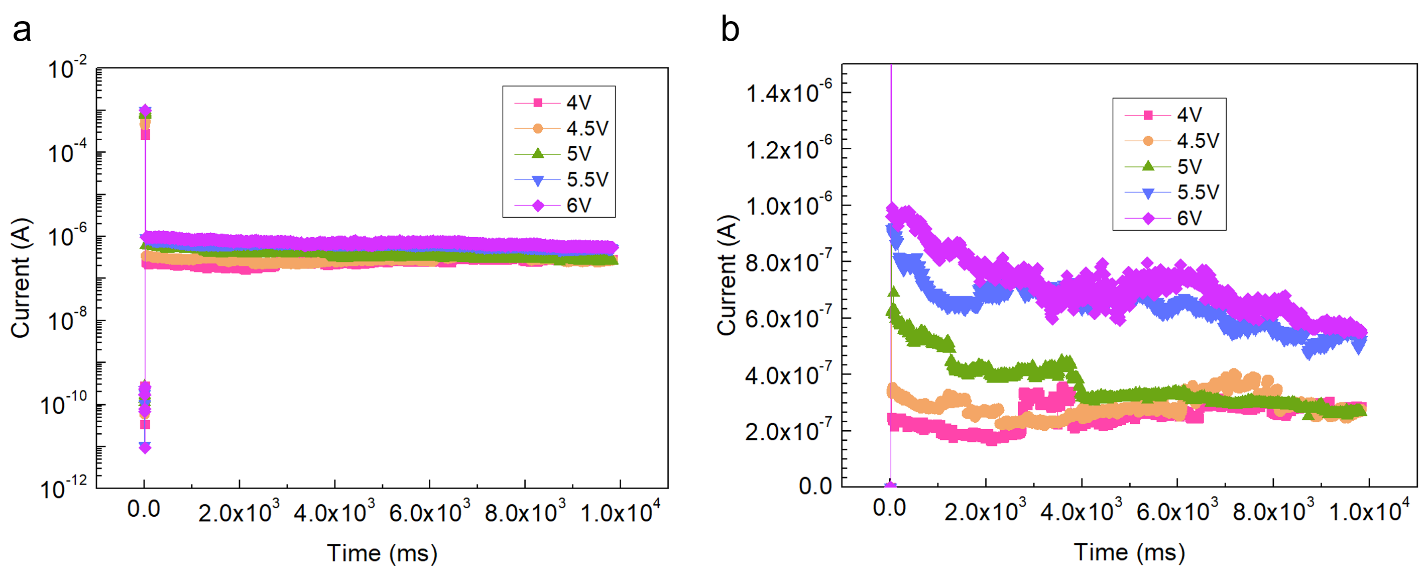


**Figure S15.** (a) Relaxation current response of a 2D-GaO_x_ nociceptor with the input voltage varied from 4 to 6V. (b) The enlarged plot of (a).


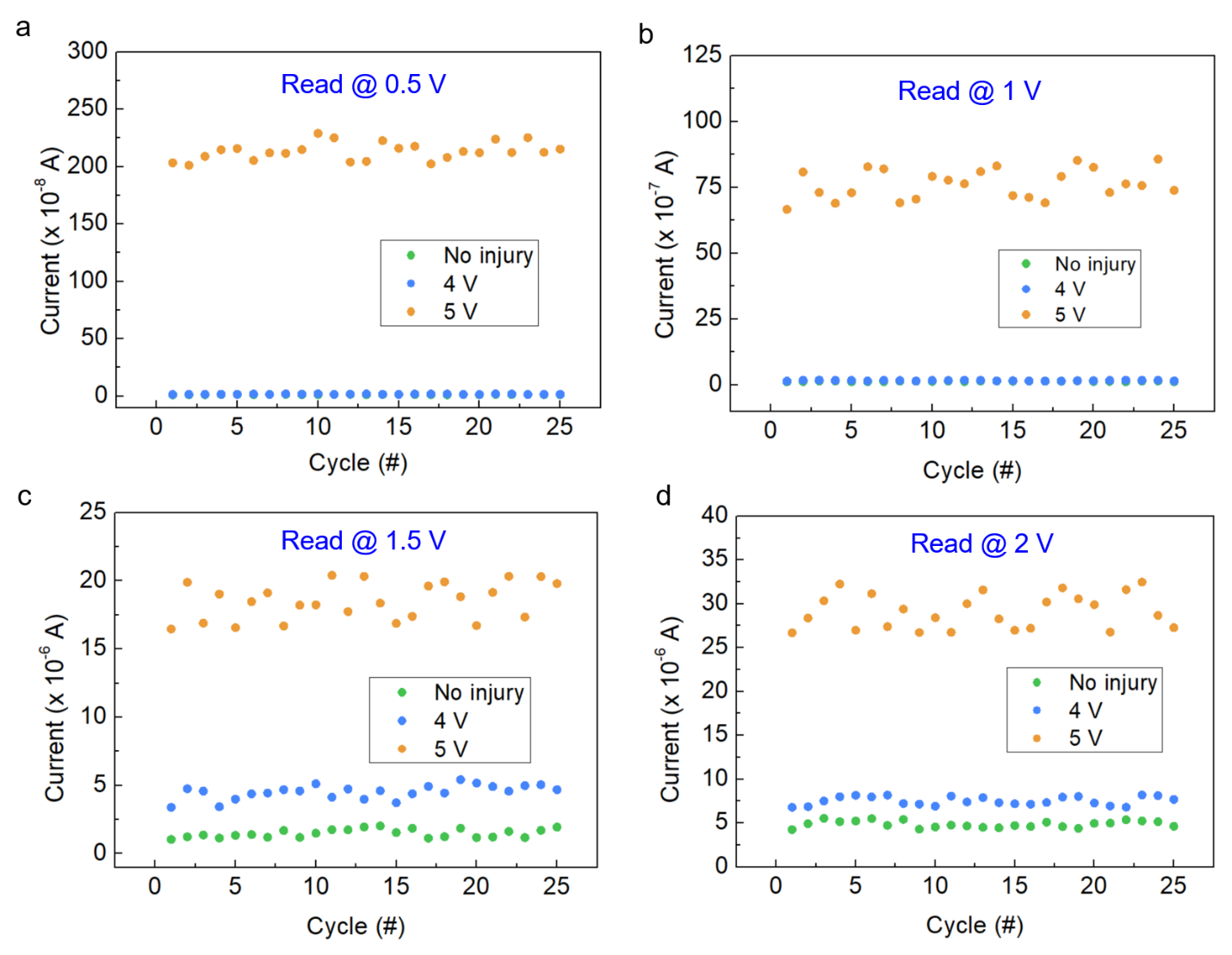


**Figure S16**. The cyclic tests of a nociceptive device with Ag/2D-GaO_x_/SiO_x_/Si structure. (a-d) The current values recorded before and after stimulus injury with a read voltage of 0.5, 1, 1.5, and 2 V, respectively.


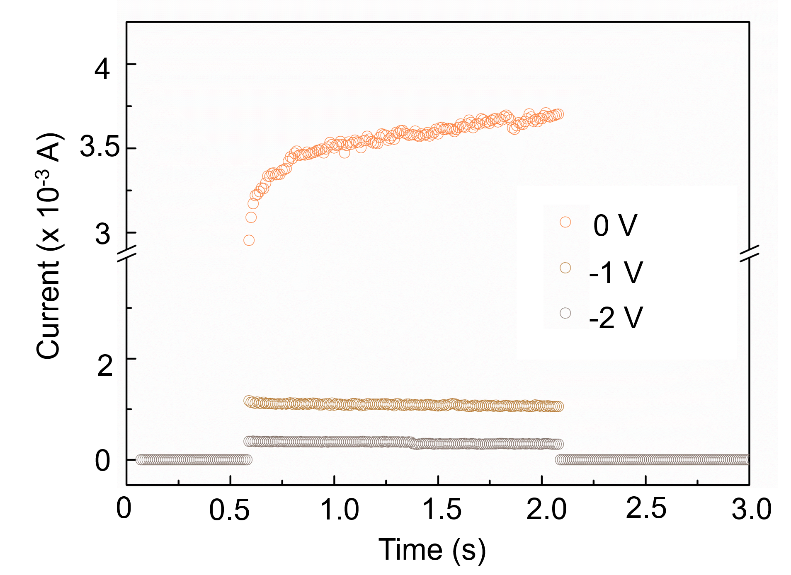


**Figure S17.** The active healing effect of a 2D-GaO_x_ nociceptor. The 2D-GaO_x_ nociceptor is subjected to an injury of 4 V with a pulse width of 5 ms, followed by a read voltage of 0.3 V. The devices are subjected to active healing bias of −1 V and −2.5 V for 5 ms, after which the sensitized responses are reread at 0.3 V.


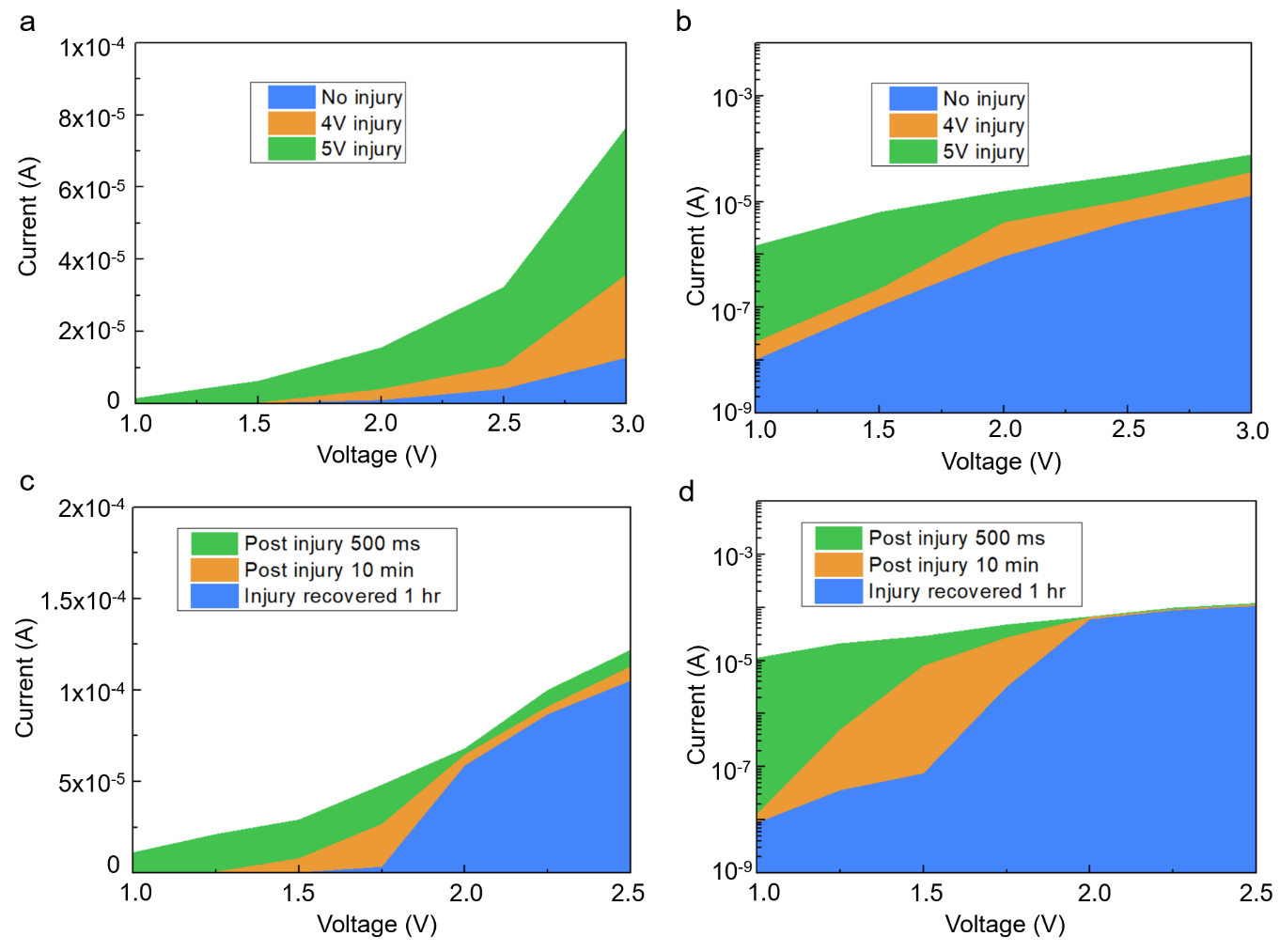


**Figure S18.** The corresponding average output currents of a 2D-GaO_x_ nociceptor at different pulse amplitudes after 6-month storage at air ambient. Average output currents for pulse amplitudes of 1, 1.5, 2, 2.5, or 3 V applied 1 min after an injury pulse of 0, 4, or 5 V, and these results on (a) linear and (b) log scales. Average output currents for pulse amplitudes of 1, 1.25, 1.5, 1.75, 2, 2.25, or 2.5 V following an injury pulse of 5 V for 25 ms with different recovery times of 500 ms, 10 min, or 1 h, and these results on (c) linear and (d) log scales.


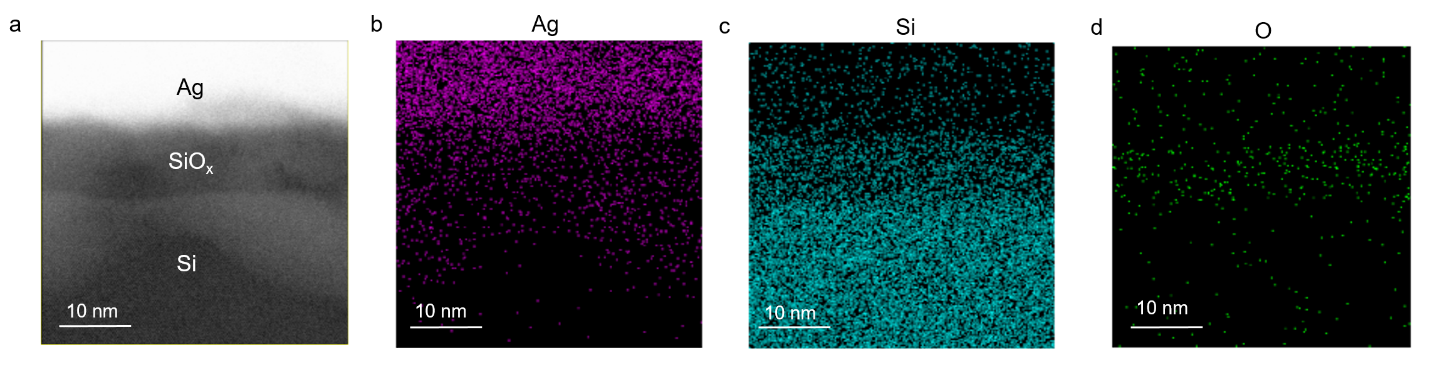


**Figure S19.** STEM-EDS elemental mapping of a device with Ag/SiO_x_/Si structure after 6V electrical stimulus. (a) Cross-sectional STEM-HAADF view of a device with Ag/SiO_x_/Si structure. (b–d) The corresponding elemental distribution of the Ag, Si, and O from EDS mappings from (a), respectively.


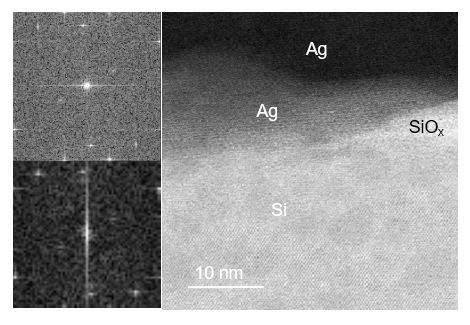


**Figure S20.** TEM image of a 2D-GaO_x_ nociceptor after consecutive 6V stimulus.


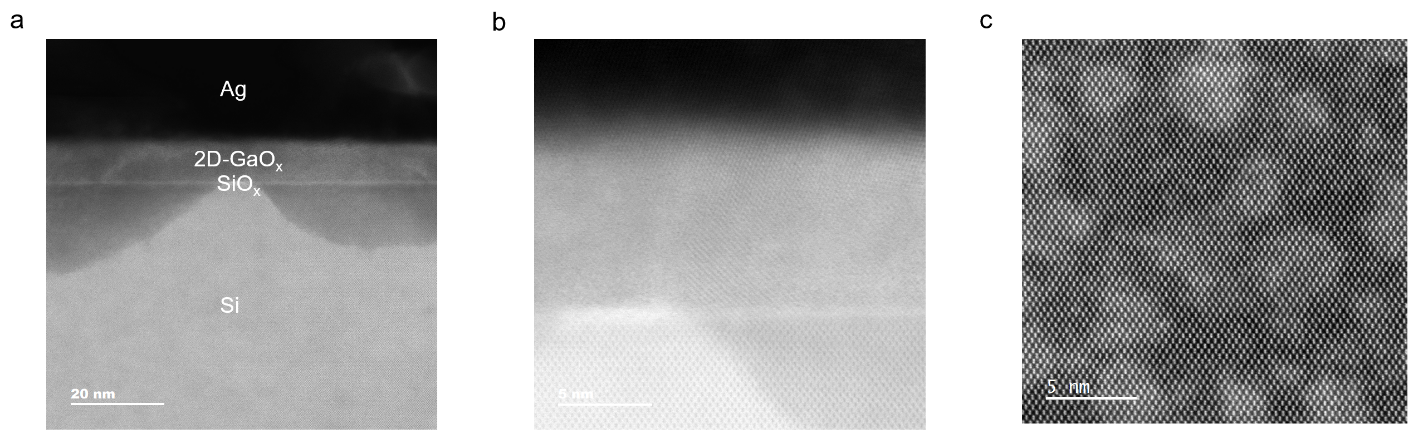


**Figure S21.** TEM images of a 2D-GaO_x_ nociceptor after 12V stimulus. (a, b) Low- and high-magnification TEM images of a 2D-GaO_x_ nociceptor with large amounts of Ag embedded in the 2D-GaO_x_ and SiO_x_, respectively. (c) TEM image of Si substrate contaminated by Ag clusters.


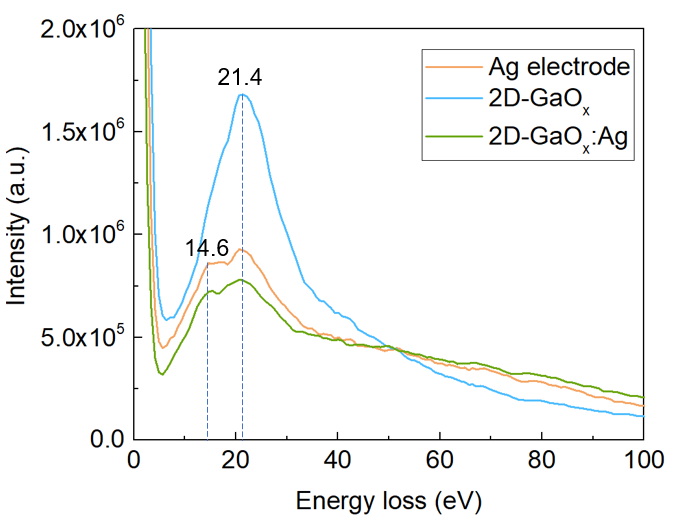


**Figure S22.** EELS low-loss spectra captured from Ag electrode (orange), 2D-GaO_x_ (blue), and 2D-GaO_x_:Ag (green) after threshold switching.
